# Injectable *Bombyx mori* (*B. mori*) silk fibroin/MXene conductive hydrogel for electrically stimulating neural stem cells into neurons for treating brain damage

**DOI:** 10.1186/s12951-024-02359-x

**Published:** 2024-03-14

**Authors:** Zhangze Yang, Yuxin You, Xiangyu Liu, Quan Wan, Zongpu Xu, Yajun Shuai, Jie Wang, Tingbiao Guo, Jiaqi Hu, Junhui Lv, Meng Zhang, Mingying Yang, Chuanbin Mao, Shuxu Yang

**Affiliations:** 1 Institute of Applied Bioresource Research, College of Animal Science, Key Laboratory of Silkworm and Bee Resource Utilization and Innovation of Zhejiang Province, Hangzhou, 310058 Zhejiang China; 2 Department of Neurosurgery, School of Medicine, Sir Run Run Shaw Hospital, Zhejiang University, Hangzhou, 310016 China; 3 Institute of Biotechnology, College of Agriculture and Biotechnology, Zhejiang University, Hangzhou, 310058 Zhejiang China; 4 School of Materials Science and Engineering, Zhejiang University, Hangzhou, 310027 Zhejiang China; 5 Centre for Optical and Electromagnetic Research National Engineering Research Center for Optical Instruments Zhejiang University, Hangzhou, 310058 China; 6 Department of Biomedical Engineering, The Chinese University of Hong Kong, Sha Tin, Hong Kong SAR

**Keywords:** Silk fibroin, Conductive hydrogel, Electrical stimulation, Neural stem cells, Traumatic brain injury

## Abstract

Brain damage is a common tissue damage caused by trauma or diseases, which can be life-threatening. Stem cell implantation is an emerging strategy treating brain damage. The stem cell is commonly embedded in a matrix material for implantation, which protects stem cell and induces cell differentiation. Cell differentiation induction by this material is decisive in the effectiveness of this treatment strategy. In this work, we present an injectable fibroin/MXene conductive hydrogel as stem cell carrier, which further enables *in-vivo* electrical stimulation upon stem cells implanted into damaged brain tissue. Cell differentiation characterization of stem cell showed high effectiveness of electrical stimulation in this system, which is comparable to pure conductive membrane. Axon growth density of the newly differentiated neurons increased by 290% and axon length by 320%. In addition, unfavored astrocyte differentiation is minimized. The therapeutic effect of this system is proved through traumatic brain injury model on rats. Combined with in vivo electrical stimulation, cavities formation is reduced after traumatic brain injury, and rat motor function recovery is significantly promoted.

**Graphical Abstract:**

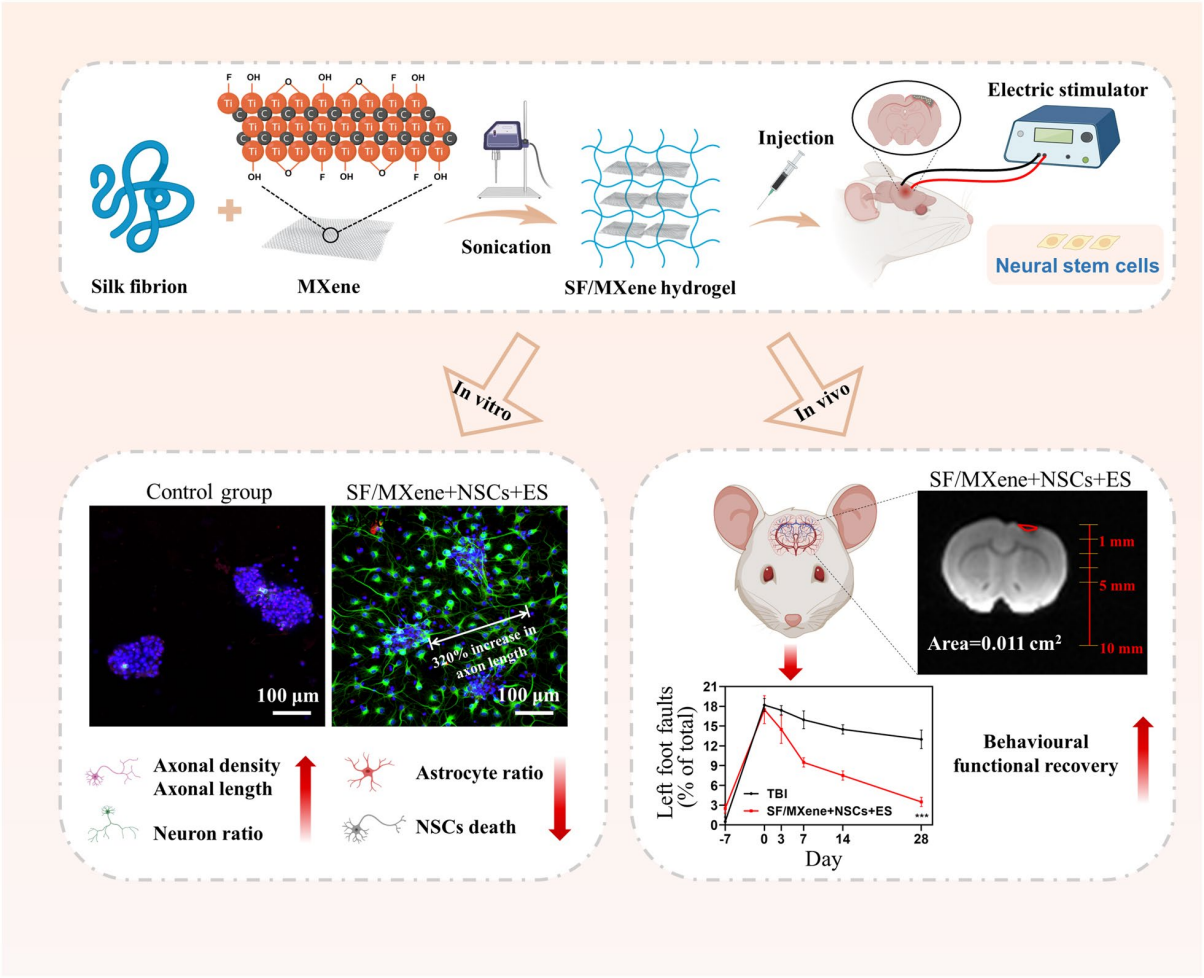

**Supplementary Information:**

The online version contains supplementary material available at 10.1186/s12951-024-02359-x.

## Introduction

Traumatic brain injury (TBI) is a common neurological disorder, that results in neurological damage that reduces the quality of life for patients [1]. At current, the clinical strategies for treating TBI mainly include surgical treatment, pharmacological treatmentand physical therapy [2]. Recent emerging neural tissue engineering therapy has shown great potential in promoting the regeneration of neural tissue [3]. The neural stem cells (NSCs), scaffold materials, and stimulation factors are essential for neural tissue engineering [4]. The scaffold materials as well as stimulation factors play an important role in supporting the proliferation and differentiation of NSCs [5]. However, the NSCs are prone to be differentiated into not only the neurons that contribute to neural repair but also the glial cells that are not conducive to repair [6]. Therefore, it is urgent to design an integration of scaffold materials and stimulation factors that can promote the differentiation of NSCs into neurons while avoiding the development of glial cells to achieve the repair and regeneration of neural tissues. Accordingly, scientists in the fields of tissue engineering are increasingly interested in how to find suitable substrates and stimulation factors to process them into ideal scaffolds for satisfying NSCs therapy by inducing NSCs differentiation [7].

Recently, numerous materials ranging from synthetic materials, including polylactic acid [8], polyglycolic acid [9], and polycaprolactone [10], to natural materials such as collagen [11], gelatin [12], and cellulose [13], have been applied in this field. A key focus has been on naturally occurring materials with biocompatibility and controllable degradation. *Bombyx mori* (*B. mori*) silk fibroin (SF), one of the natural biomaterials, has been considered a particularly promising candidate by having exceptional mechanical properties, excellent biocompatibility, and controllable biodegradability [14]. The tunable mechanical properties of the SF hydrogels can match the physiological stiffness of the brain tissue [15]. Furthermore, the sol-to-gel transition of SF solutions via simple, chemical-free sonication offers a clear advantage for potential clinical applications, simplifying the process when translating these systems to practical applications [16]. Many studies have proven that SF hydrogel can simulate extracellular matrix (ECM) microenvironments in the field of tissue engineering [17, 18]. For instance, Cheng reported that the SF/silica nanoparticles hydrogel exhibited excellent biocompatibility properties and osteogenic properties for the treatment of cranial defects [19]. Our group has successfully developed various SF-based microparticle platforms capable of incorporating both antigen and adjuvant for cancer immunotherapy [20]. Recently, we designed SF microparticles loaded with bacteriophages that can promote the regeneration of stroke-damaged brain tissue [21]. Natalia Gorenkova et al. also pointed out that SF hydrogel exhibited excellent spatial conformity and biocompatibility in stroke treatment, further confirming that SF hydrogel can serve as a promising material for neural repair scaffolds [22]. However, there are still challenges in how to induce stimulation factors for promoting the directed differentiation of NSCs into neurons.

As we know, bioelectricity significantly affects the natural NSCs differentiation of the human brain, implying that conductive materials can be used as simulation factors for neural tissue engineering [23]. Therefore, it has been reported that the combination of conductive materials such as germanium phosphide (GeP) [24], carbon nanotubes (CNT) [25], and reduced graphene oxide [26], into hydrogels can generate electrical conductivity to simulate the differentiation of NSCs into neurons. For instance, Jong Min Lee et al. proved that the polyethylene glycol/silver nanowire (AgNW) conductive hydrogels could direct the differentiation of NSCs [27]. MXene, one of the two-dimensional transition metal carbides and nitrides, has been attracting interest due to its high electrical conductivity, good biocompatibility, high mechanical properties, and modifiable characteristics [28]. While a range of conductive materials such as the graphene family, CNT, and black phosphorus exist, their practical use is often constrained [29]. The limited stability and water solubility of graphene, CNTs, and black phosphorus hinder their ability to bind with other materials [30, 31]. Conversely, MXene stands out due to its surface-adjustable functional groups. These functional groups not only reduce its oxidation upon storage but also enhance its water dispersibility and stability [32]. This facilitates the formation of diverse composites and microstructures through combination with other biomaterials. Recently, increasing research has focused on the effects of MXene on neural repair [33]. Guo et al. found that the MXene membrane combined with electrical stimulation (ES) could promote NSCs neural differentiation [34]. This suggests that the combination of MXene and SF is an excellent material system for stimulating NSCs differentiation into neurons.

**Scheme 1.**
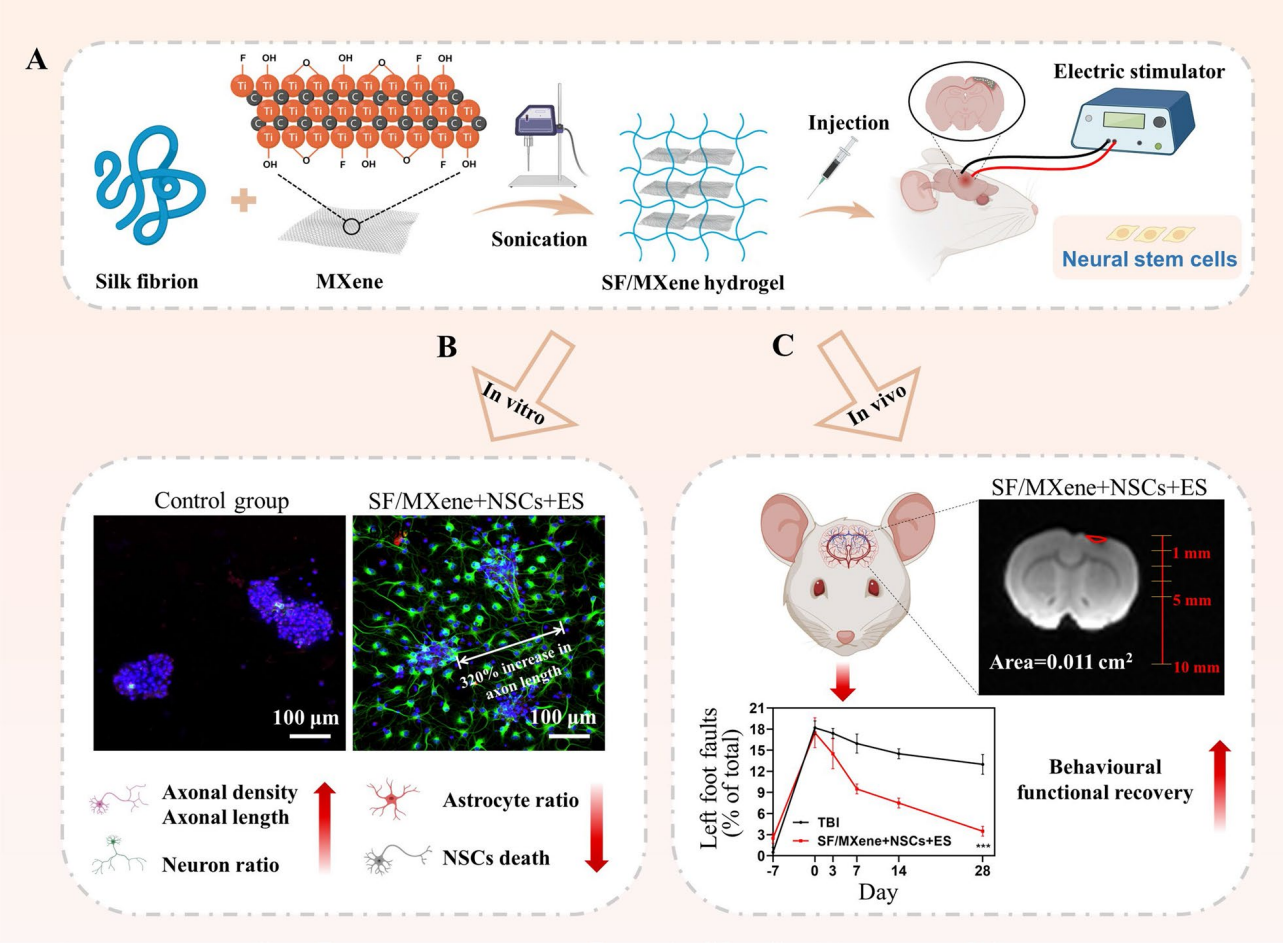
Schematic illustration. **A** Preparation process of SF/MXene hydrogel. **B** Images of NSCs neural differentiation on SF/MXene hydrogel with ES in vitro. **C** SF/MXene hydrogel combined NSCs with ES to treat TBI

Therefore, we combined SF and MXene to prepare a hydrogel scaffold and induce the differentiation of NSCs into neurons with ES, resulting in a 320% increase in axonal length while reducing the number of glial cells to 14%. Firstly, we added MXene dispersion to the SF solution and obtained SF/MXene hydrogels by sonication (Scheme 1). The SF/MXene hydrogel forms an interconnecting network through intermolecular electrostatic and hydrogen bonding interactions Then, we tested the hydrogels for injectable, mechanical, and electrical properties to ensure they meet the requirements for implantation in the brain. In vitro NSCs cultures showed that SF/MXene hydrogel exhibited excellent biocompatibility, neural differentiation ability, and axonal-directed regeneration ability. In addition, we constructed traumatic brain injury (TBI) models to assess the neural regeneration capacity by in situ implantation of SF/MXene hydrogel loaded with NSCs. Furthermore, implantation of the NSCs-SF/MXene hydrogel could reduce the area of the cavity and promote neuronal differentiation after TBI, as well as reduce the accumulation of astrocytes and glial scar, ultimately promoting the recovery of motor function in SD rats. This new hydrogel system is the first attempt to use SF and MXene to make hydrogel for NSCs-ES combination therapy and shows great potential for application in clinical brain damage treatment.

## Materials and methods

### SF solution preparation

Firstly, *B. mori* cocoons were boiled in 0.02 mol L^−1^ Na_2_CO_3_ solution for 30 min to remove sericin. Secondly, the SF fibers were dissolved in 9.3 mol L^−1^ LiBr solution at 60 °C for 4 h. The solution was dialyzed with deionized water for 96 h. Finally, the solution was centrifuged at 3000 rpm at 4 °C for 20 min to remove any silk aggregates. The resulting silk solution was stored at 4 °C until use [35].

### Preparation of self-assembling SF/MXene (Ti_3_C_2_T_x_) conductive hydrogels

The silk solution was sonicated using an ultrasonic cell crusher to prepare the SF hydrogel. Typically, the 20 mg mL^−1^ SF solution was exposed at 15% amplitude to 12 sonication cycles, each consisting of 15 s on and 5 s off, inducing the solution-gel transition. To prepare the SF/MXene hydrogels, we mixed the MXene (Ti_3_C_2_T_x_) colloidal solution into the SF solution at concentrations of 0.66 wt% (SF/MXene = 150:1), 1 wt% (SF/MXene = 100:1), and 2 wt% (SF/MXene = 50:1) of MXene, respectively. Then, these SF/MXene solutions were homogeneously mixed and sonicated to form hydrogels. Subsequently, the resulting SF/MXene hydrogels were denoted as SF/MXene-0.66%, SF/MXene-1%, and SF/MXene-2%, respectively.

### Characterization of SF/MXene hydrogels

The morphology of SF/MXene hydrogels was observed by using a scanning electron microscope instrument (SEM). Fourier transform infrared (FTIR) spectroscopy of the SF/MXene hydrogels was tested to analyze the structural characteristics. The titanium (Ti) element content in the SF/MXene hydrogel was analyzed using an X-ray Fluorescence Spectrometer (XRF). The swelling properties were measured through a water absorption test. The rheological characterization of SF/MXene hydrogels was performed using a rotational rheometer. Mechanical properties were evaluated via compression tests using an electronic universal testing machine. Furthermore, conductivity measurements were performed using the four-probe method. The electrochemical impedance spectroscopy (EIS) measurements of SF/MXene hydrogels were performed using the electrochemical workstation.

### Biocompatibility evaluation of the SF/MXene hydrogels

NSCs (Cyagen, China) were cultured on SF/MXene hydrogels. Cell viability was assessed using the CCK-8 assay (Beyotime, China) and live/dead staining assay (Beyotime, China) on days 1, 3, and 5. Cells planted on SF hydrogels and cell culture plates were used as controls. For 3D cell culture, NSCs were loaded inside SF/MXene hydrogels at 2 × 10^6^ cells mL^−1^ density. These NSCs-loaded SF/MXene hydrogels were collected to perform live/dead assay on days 1, 3, and 5.

### Cell proliferation under different ES conditions

CCK-8 assay and live/dead staining assay were used to analyze cell proliferation. NSCs were plated on the surface of the pre-treated SF/MXene hydrogel after passaging at a concentration of 2 × 10^5^ cells mL^−1^. The hydrogels were stimulated with different voltage strengths for 30 min every 24 h starting from the second day. Briefly, the 5 mm platinum electrodes were sterilized under 75% ethanol and UV light. The SF/MXene hydrogel was prepared in a 24-well plate, and platinum electrodes were inserted into the hydrogel to generate an electric field inside it. At the end of ES, the cells were incubated for another 12 h. The different voltage groups were examined using the CCK-8 assay. On day 5, the samples were fixed in 4% paraformaldehyde for 30 min and washed with PBS. After fixation, the cytoskeletons were stained with live/dead staining assay on day 5. As for qRT-PCR, total RNA was collected using the RNA extraction kit (Vazyme, China) on days 3 and 10. The RNA samples were reverse transcribed into cDNA using a reverse transcription kit (Vazyme, China). SYBR qPCR master mix™ (Vazyme, China) was used to amplify the target gene. Glyceraldehyde-3-phosphate dehydrogenase (GAPDH) was used as an internal reference gene. The relative quantification method was used to compare the differences in transcript expression of target genes between groups. The primer sequences are shown in Additional file 1: Table S2.

### In vitro differentiation of NSCs on SF/MXene hydrogel

To investigate NSCs' differentiation potential, we cultured them on SF/MXene hydrogels with and without ES. Immunocytochemistry, RT-qPCR analysis, and RNA-sequencing were then performed. On days 3 and 10, the cells were harvested and incubated with primary antibodies for neuron marker β-tubulin III (1:100, HuaAn, China) and astrocyte marker GFAP (1:100, HuaAn, China). Subsequently, fluorescent secondary antibodies (1:500, HuaAn, China) were added, and DAPI was used to stain the cell nuclei prior to imaging. For qRT-PCR analysis, total RNA was collected on days 3 and 10 as previously described. For RNA-sequencing, total RNA was extracted by using the TRIzol reagent (Beyotime, China). The purity and concentration of RNA were then evaluated, along with an assessment of RNA integrity. Finally, the transcriptome sequencing was carried out by Shanghai Majorbio Bio-pharm Technology.

### Establishment of an ES model of the TBI animal model

Male SD rats (250 g) from Zhejiang University Experimental Animal Center were used for the experiment, following the guidelines of the Animal Protection and Ethics Committee of Zhejiang University. We established the TBI rat model using the modified Feeney’s weight-drop method [36]. After inducing anesthesia, the rats were shaved and fixed in a prone position. Then, the rats were fixed prone and the scalp was incised along the midline to expose the right skull. A diameter bone window was opened at 2 mm to the right of the midline and 3 mm behind the anterior fontanel using a skull bore device. Care should be taken to protect the dura mater while drilling the skull. Next, the rats were fixed on the stereotaxic instruments so that the percussion firing pin was aligned with the bone window. The percussion pin was slowly lowered 2 mm after contact with the dura mater. A hammer was used to impact the firing pin from a height of 20 cm in a free fall, causing moderate traumatic brain injury. After the impact, the wound was quickly cleaned. Finally, the bone window was closed with bone wax, and the scalp was sutured. After the operation, we maintained the body temperature at 37 °C using a thermal insulation pad until the rats woke up. Additionally, we injected 1 mL kg^−1^ of penicillin intraperitoneally to prevent infection.

After the TBI model was established, rats were randomly divided into the following five groups: TBI group (Blank control), and SF group (SF hydrogel). SFM group (SF/MXene hydrogel), SFMC group (SF/MXene hydrogel + NSCs), and SFMC + E group (SF/MXene hydrogel + NSCs + ES). In the SFMC + E group, rats were implanted with chronic bipolar stimulation electrodes following the implantation of hydrogels mixed with NSCs. Subsequently, ES was applied to promote TBI repair. The electrode wires had beveled tips for easy insertion into the hydrogel, and the electrodes were subsequently fixed to the skull with dental cement. Notably, the electrodes were tailed with indestructible rows of female connectors suitable for long-term implantation, allowing stimulation experiments to be accomplished with the rats freely moving. The proposed electric current from the stimulator can be transmitted through the electrodes to apply a pro-differentiation electric field to the NSCs inside the SF/MXene hydrogel. Throughout the entire duration of the ES treatment, the rats did not exhibit any significant agitation or discomfort.

### Nuclear magnetic resonance examination

After 28 d of treatment, brain tissue was taken and fixed with 4% paraformaldehyde. Each brain was fixed in a clinical 3T NMR scanner to observe the volume of the injured tissue. Imaging was performed with a T_2_-weighted fast spin-echo imaging sequence (T_R_/T_E_ = 2000/55 ms, slice thickness = 1.3 mm). An experienced radiologist then evaluated the imaging. The volume of the injured tissue was then measured using RadiAnt DICOM Viewer and Image J software.

### Immunofluorescence staining

Immunofluorescence staining was performed to assess the expression of specific markers in brain tissue sections from rats 28 days after transplantation treatment. The expression of the β-tubulin III, and GFAP in the damaged area was detected. The expression of neuronal marker NeuN and cell proliferation marker Ki67 in the Dentate gyrus (DG) region was also measured. Brain tissues were first fixed with 4% paraformaldehyde. Paraffin embedding was then performed. After paraffin sections were dewaxed, the sections were added to the EDTA repair solution and closed using donkey serum. The blocking solution was aspirated and washed 3 times with PBS for 5 min each. Sections were incubated with primary antibody at 37 °C for 2 h. The primary antibody was aspirated and washed 3 times with PBS for 5 min each time. The fluorescent secondary antibody was incubated at 37 °C for 40 min, aspirated, and washed 3 times with PBS for 5 min each.

### The blood–brain barrier (BBB) integrity test

BBB permeability was assessed by measuring the concentration of Evans Blue dye in the rat brain. Evans Blue was prepared as a 2% solution in PBS. Rats were anesthetized and injected with Evans Blue (4 mL kg^−1^) via the tail vein. After complete blood replacement with saline, the rat brains were quickly removed. The left and right hemispheres were separated and soaked in 2 mL formamide at room temperature for 48 h. The absorbance of the supernatant was measured at 625 nm. The remaining brain tissue was dried at 80 °C for 3 days. The results were expressed as relative absorbance (unit g^−1^ dry weight).

### Assessment of animal behavior test

To evaluate the sensorimotor function of SD rats, we used modified neurologic severity scores (mNSS), sucrose preference test (SPT), cylinder test, and grid-walking test. The mNSS test was carried out on 3, 7, 14, 21, and 28 d after the hydrogel injection. Higher scores mean more severe neurological deficits, ranging from 0 (Healthy) to 18 (Most severe). Sensorimotor functions were measured by the mNSS, cylinder test, and grid-walking test. We also used the SPT to detect anxiety or depression symptoms in animals, as a lower sucrose preference index indicates the presence of a lack of pleasure, a significant symptom of depression.

### Statistical analysis

All these data were expressed as mean ± standard deviation. One-way ANOVA was performed using Prism 8.0 (GraphPad). Differences between groups were considered statistically significant when *P < 0.05, **P < 0.01, and ***P < 0.001. If the *P* value was greater than 0.05, the results were considered not statistically different.

## Results and discussion

### Preparation and screening of SF/MXene hydrogels

**Fig. 1:**
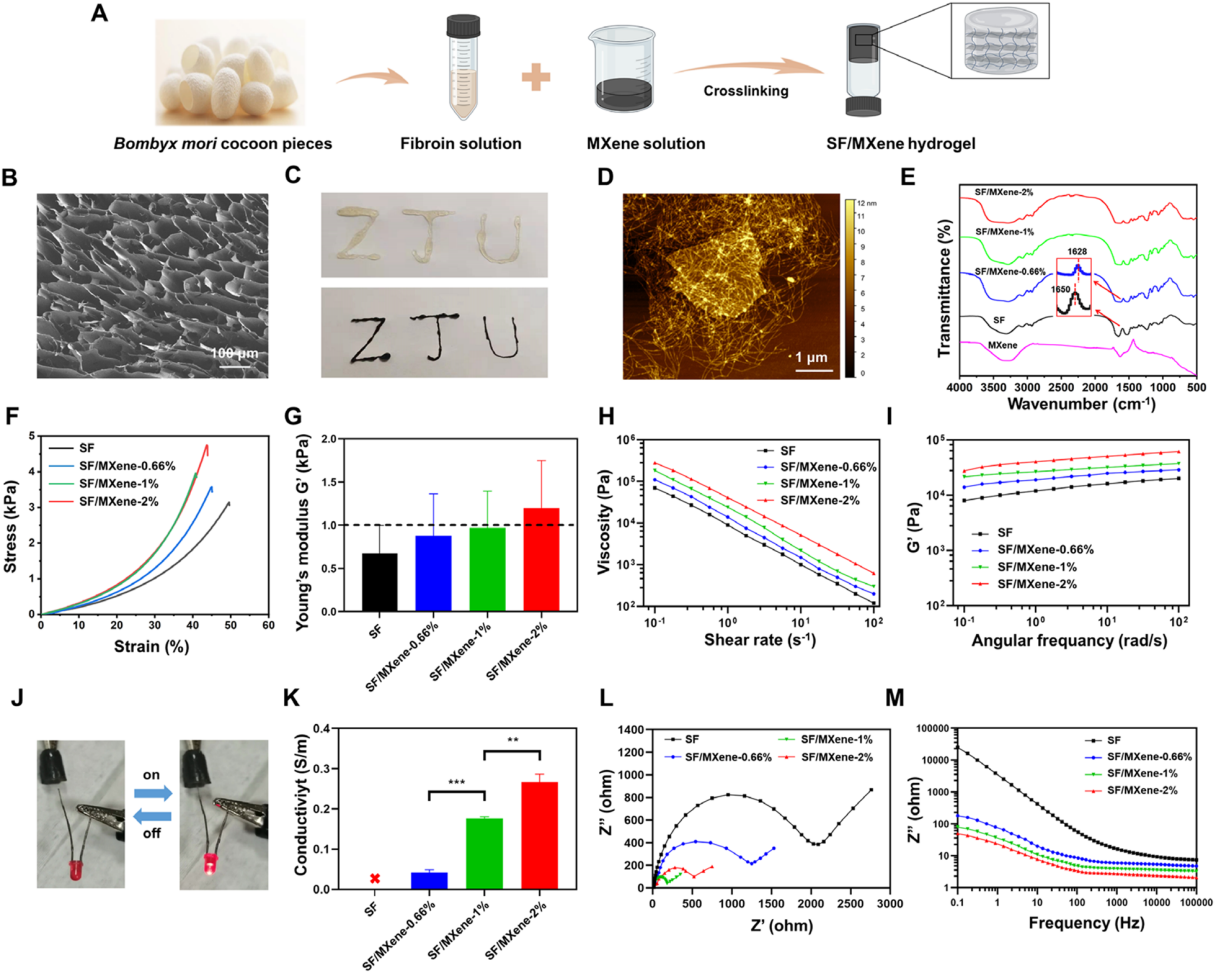
Characteristics of the SF-based hydrogels with varying MXene content. **A** Schematic illustration of the SF/MXene hydrogel preparation process by sonication. **B** SEM image of SF/MXene-1% hydrogel. **C** “ZJU” written by the SF/MXene-1% hydrogel through a 26-gauge needle **D** AFM morphology of SF/MXene-1% hydrogel. **E** FTIR spectra of SF-based hydrogels with varying MXene content. **F** Compressive stress–strain curves and **G** Young’s modulus of SF-based hydrogels with varying MXene content. **H** Storage modulus G’ of SF-based hydrogels with varying MXene content. **I** The viscosity of SF and SF/MXene hydrogels with the shear rate from 0.1 to 100 s^−1^. **J** The state of a light-emitting diode in an SF/MXene-1% hydrogel-connected circuit. **K** The conductivity of the SF-based hydrogels with varying MXene content. **L** Nyquist curves and (M) impedance curves of SF-based hydrogels with varying MXene content. (± SD, n = 3; **P < 0.01; ***P < 0.001)

SF and MXene were mixed in different concentration ratios and cross-linked by ultrasonication to obtain SF/MXene hydrogels (Fig. 1A). Figure 1B indicates the typical internal structure of SF/MXene-1% hydrogel. We observed that the prepared hydrogels have a large pore size, which promotes nutrient infiltration, oxygen, and carbon dioxide exchange, and promotes the proliferation and differentiation of NSCs. Figure 1C showed that the letters “ZJU” were injected through a 26-gauge needle, proving SF/MXene-1% hydrogel has good injectable properties. We characterized the formation process of this structure through AFM and FTIR. Figure 1D presents that the SF self-assemble into densely arranged nanofibers on MXene nanosheets. This is mainly due to the presence of oxygen-containing functional groups on the surface of MXene nanosheets, which would lead to the binding of SF to MXene nanosheets. Self-assembly of nanofibers contributes to its porous structure and mechanical properties [37], proving that it can meet the requirements of the hydrogel for porosity and mechanical properties.

The secondary structure changes in the self-assembly process are shown in Fig. 1E. Peaks at 3425, 1626, and 535 cm^−1^ confirmed the presence of MXene. Blue shifting of SF peaks at 1628, 1530 cm^−1^ in comparison to pure SF sample, represents the conversion to β-sheets secondary structure [16]. The XRF results confirmed the presence of titanium in the SF/MXene hydrogels (Additional file 1: Table S1). The titanium content in the SF/MXene-2% group was 1.14%, which is higher than that in the SF/MXene-1% group (0.62%) and the SF/MXene-0.66% group (0.42%). Figure 1F showed the compressive stress gradually increased with the increase of MXene concentration. The Young's moduli of SF, SF/MXene-0.66%, SF/MXene-1%, and SF/MXene-2% hydrogels were 589, 746, 921, and 1284 Pa, respectively (Fig. 1G). These results indicated that the mechanical properties of SF/MXene hydrogels can be slightly altered by controlling the ratio of MXene to SF. Generally, the elastic modulus of brain tissue ranges from 300 to 1500 Pa, with an average value of 1000 Pa [38]. Furthermore, the SF/MXene hydrogels were able to maintain a Young’s modulus similar to that before syringe injection (Additional file 1: Fig. S1). Therefore, the prepared SF/MXene hydrogels can provide appropriate mechanical support for NSCs. As shown in Fig. 1H, the viscosity gradually decreased as the shear rate increased, indicating that the SF/MXene hydrogels had shear-thinning characteristics, which are necessary for the continuous flow of injectable hydrogel. Additionally, Fig. 1I and Additional file 1: Fig. S2 showed the hydrogel crosslinking system is validated by the significant increase in the G’/G” ratio. In our study, the SF/MXene hydrogels did not show significant swelling (Additional file 1: Fig. S3). This phenomenon can be ascribed to two factors: (a) The decrease in swelling ratio might be due to the MXene nanosheets providing more hydroxyl groups to be crosslinked with the amino acid chain of SF. (b) Moreover, the decrease is related to the smaller average pore size of the hydrogel, which ultimately leads to a decrease in the total water content. These results showed that the porous hydrogel formed by the self-assembly process meets the requirements in terms of morphology and mechanical properties. The mechanical properties are correlated with the structural state of the material and its swelling behavior. This is due to the fact that stronger crosslinking of the SF/MXene hydrogel not only weakens the ability to absorb water but also enhances its mechanical strength. Such a phenomenon is also commonly observed in other hydrogels [39, 40].

Electrophysiological properties and bioelectrical signaling behavior of neural tissue play a crucial role in maintaining nerve cell growth and neural tissue development [24]. The pure SF hydrogel is almost non-conductive (Fig. 1K). As the MXene content in the SF/MXene hydrogel increased to 0.66 wt%, its electrical conductivity reached 0.29 S m^−1^, indicating good electrical activity. The conductivity of the SF/MXene-1% (0.18 S m^−1^) and SF/MXene-2% (0.29 S m^−1^) hydrogels are close to that of the central nervous system tissue under physiological conditions (0.1–1 S m^−1^). These results suggest that increasing the MXene content enhances the conductivity of the SF/MXene hydrogels, which enhances the electrical signal exchange and conduction between the hydrogel and cells. As the MXene concentration in the hydrogel increased from 0 to 2%, the Nyquist curve showed a gradual decrease in the semicircle diameter. This trend suggests that SF/MXene hydrogels exhibit lower charge transfer resistance and improved electrical properties. Moreover, the slope of arc tangent in the high-frequency region is steeper, indicating that SF/MXene hydrogels possess a reduced interfacial charge transfer impedance, and therefore have a higher carrier transfer rate, which is beneficial for the ES process [41]. Consistent with the Nyquist curve, the Bode plot also confirms the lower impedance of SF/MXene hydrogels (Fig. 1L). This reduction in interface resistance facilitates communication between nerve cells via bioelectrical signaling, consequently enhancing nerve cell growth and neural tissue regeneration and development [42]. As previously introduced, conductivity plays a crucial role in transmitting electrical signals. Consequently, based on the aforementioned test results, we selected SF/MXene-1% hydrogel to validate its current loop, as illustrated in Fig. 1J.

**Fig. 2:**
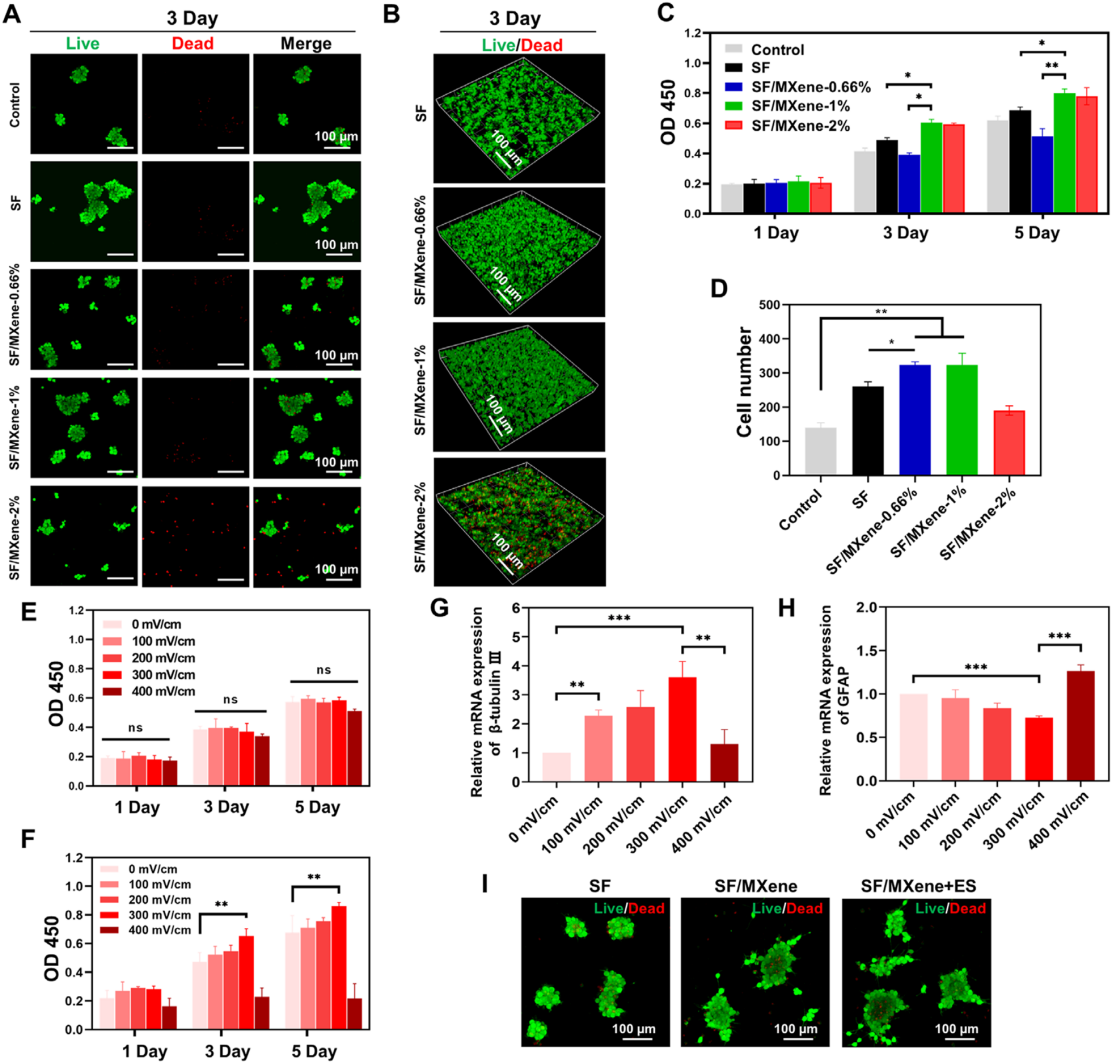
In vitro cytotoxicity of SF/MXene hydrogel and electrostimulation of NSCs on SF/MXene hydrogel with different voltages. **A** Live/dead cellular staining of the NSCs seeded on the surface of SF/MXene hydrogels containing different concentrations of MXene on day 3. The control group cells were directly seeded onto the surface of the tissue culture plate. Live cells were stained green, and dead cells were stained red. **B** 3D view of NSCs in SF/MXene hydrogels containing different concentrations of MXene on day 3. **C** Statistics of adherent cells in each group on day 5. **D** Proliferation ability of NSCs after being cultured on the surface of SF/MXene-1% hydrogels containing different concentrations of MXene for 1, 3, and 5 days. **E** Proliferation of NSCs on SF hydrogel and **F** SF/MXene hydrogel on 1, 3, and 5 d after ES with different voltages. **G** The gene expression levels of neurons (β-tubulin III) and **H** astrocyte (GFAP) were analyzed on day 5. **I** Live/dead cellular staining of the NSCs in SF, SF/MXene, SF/MXene + ES (300 mV cm^−1^) groups on day 5. (± SD, n = 3; *P < 0.05; **P < 0.01; ***P < 0.001)

The biocompatibility of SF/MXene hydrogels was tested by the commonly used live/dead staining and CCK-8 assay, and the results are as follows. As shown in Fig. 2A, the density of dead cells increased as the concentration of MXene increased to SF/MXene-2%. In contrast, both SF/MXene-0.66%, and SF/MXene-1% hydrogels showed good biocompatibility and more cell adhesion than the SF group. To further evaluate the biocompatibility of the SF/MXene hydrogels, we used the confocal microscope 3D scanning function (Fig. 2B). It can be seen that the SF, SF/MXene-0.66%, and SF/MXene-1% hydrogels have good NSCs compatibility, which satisfies the cell transplantation requirements for subsequent animal experiments. Moreover, the proliferation capacity of NSCs on SF/MXene hydrogels was studied. NSCs exhibited a higher cell number on both SF/MXene-1% and SF/MXene-0.66% hydrogels than the SF group, indicating good cytocompatibility and enhanced cell proliferation with MXene incorporation (Fig. 2C and D). The presence of MXene increased hydrogel surface roughness, leading to elevated cellular tension and enhanced cell proliferation, in line with prior research [43]. Considering the mechanical properties, electrical conductivity, cytocompatibility, and cell adhesion, SF/MXene-1% hydrogel was selected for future experiments.

The purpose of performing ES is to induce the differentiation of NSCs towards neurons that are conducive to injury repair. Therefore, it is important to find the appropriate voltage. To investigate the effect of ES at different voltages, the proliferation and differentiation of NSCs on SF and SF/MXene hydrogels were examined. When the voltage was applied to the SF hydrogel, no significant effect on cell activity was found due to its non-conductive nature, with the applied voltage not acting on the cells (Fig. 2E). As shown in Fig. 2F, significant differences in the number of cells that occurred in the different voltage groups can be seen. The results showed that the highest OD value was obtained when ES treatment was performed at 300 mV cm^−1^ on day 5. Similarly, Wang reported that appropriate ES can enhance the proliferative activity of NSCs, while excessive ES can inhibit cell proliferation [44]. Moreover, RT-qPCR analysis demonstrated that after 5 days of ES, various voltage intensities positively influenced neuronal differentiation. β-tubulin III, a microtubule element found primarily in neurons, is a specific marker for neuronal cells. Glial fibrillary acidic protein (GFAP) is a marker for astrocytes, which are a type of glial cell in the central nervous system. Therefore, gene expression levels of β-tubulin III and GFAP are commonly used indicators of neural differentiation. The continuous activation and excessive proliferation of astrocytes after neural repair can lead to the formation of glial scars, which can prevent the regeneration of newborn neurons. Figure 2G showed the 300 mV cm^−1^ group exhibited the best neuronal differentiation effect. In contrast, ES had inhibitory effects on the differentiation of astrocytes, with the 300 mV cm^−1^ group showing the most pronounced inhibitory effect (Fig. 2H). Based on the above results, we chose 300 mV cm^−1^ as the most suitable ES condition for the differentiation of NSCs. Furthermore, live/dead staining was used to detect the viability of NSCs on day 5 after applying optimal ES to the hydrogels (Fig. 2I). Additional file 1: Fig. S4 revealed that the SF/MXene + ES (300 mV cm^−1^) group had the highest viable cell rate (92.1%), followed by SF/MXene (87.8%) and SF (78.5%) groups.

### In vitro differentiation validation of NSCs

**Fig. 3:**
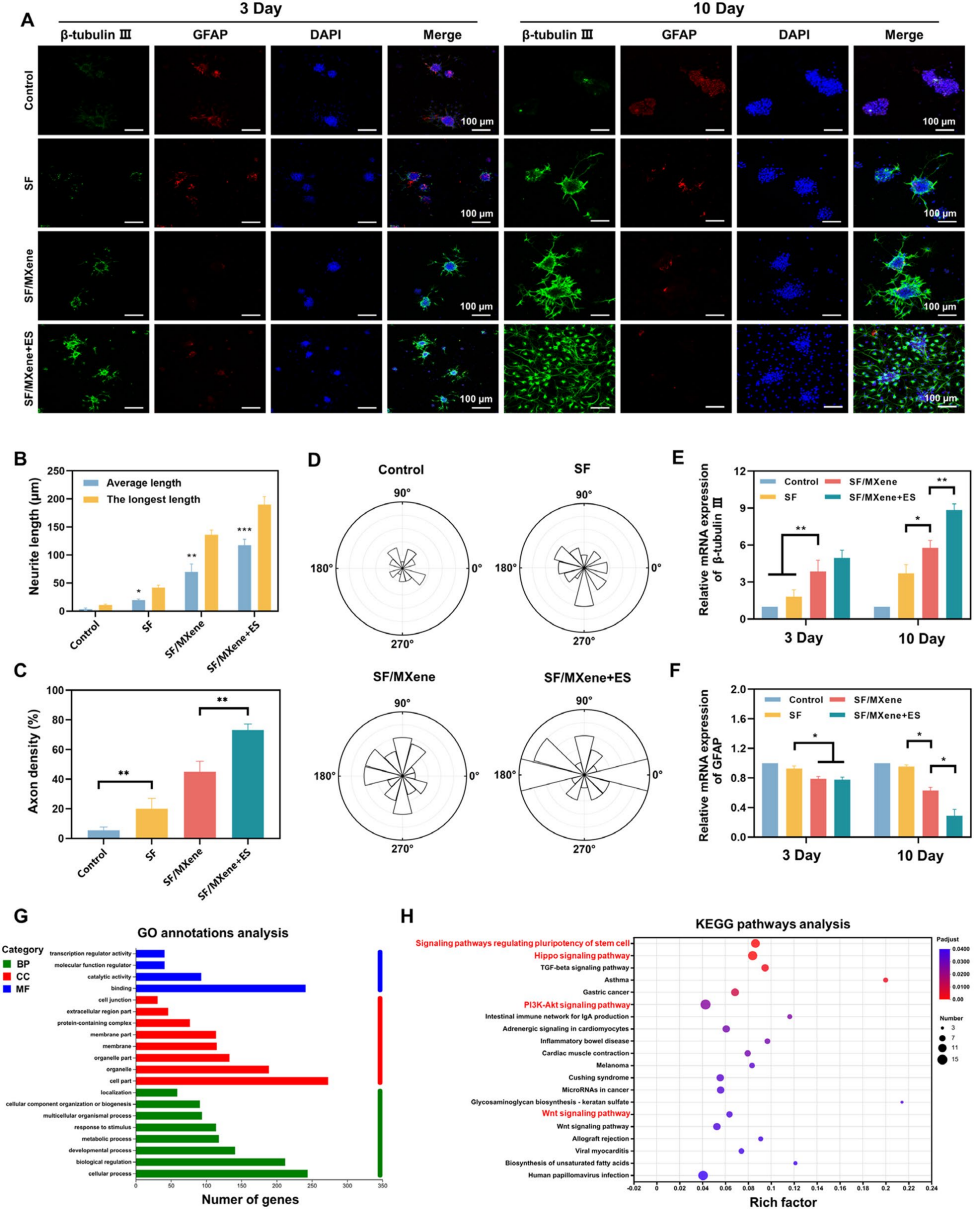
The differentiation of NSCs with the SF-based hydrogels in vitro. **A** Immunofluorescent staining of NSCs showing β-tubulin III-positive cells (green) and GFAP-positive cells (red) in different groups on days 3 and 10. The control group cells were directly seeded onto the surface of the tissue culture plate. **B** Statistics of the average axon length and longest axon length of each group. **C** Statistics of axon density of each group. **D** Rose wind diagram of axon distribution for each group. Each sector represents the number of axons within the corresponding angular range, with a larger sector area indicating a higher number of axons distributed at that angle. **E** Gene levels of β-tubulin III and **F** GFAP of the NSCs differentiation with the prepared hydrogels on days 3 and 10. **G** Categorize the differentially expressed genes into biological process (BP), cellular component (CC), and molecular function (MF) groups according to the Gene Ontology (GO) classification. **H** Enriched KEGG pathway analysis according to differentially expressed genes between the SF/MXene + ES and SF groups. (± SD, n = 3; *P < 0.05; **P < 0.01; ***P < 0.001)

As shown in Fig. 3A, NSCs were more likely to differentiate into neurons on softer hydrogel substrates compared to the control group. The expression of β-tubulin III was significantly elevated in cells cultured on SF/MXene hydrogels compared to those cultured on SF hydrogels. In contrast, the expression level of the GFAP was reduced on SF/MXene hydrogels. The combined use of SF/MXene hydrogel and ES resulted in the highest proportion of β-tubulin III-positive cells and the lowest proportion of GFAP-positive cells, indicating that ES further promoted the differentiation of NSCs into neurons while inhibiting their differentiation into astrocytes (Additional file 1: Fig. S5A and B). From day 3 to 10, β-tubulin III expression increased significantly in all groups. On day 10, the mean fluorescence intensity of the SF/MXene + ES group was 8.16, which was significantly higher than that of the control (3.89), SF (4.48), and SF/MXene (6.21) groups (Additional file 1: Fig. S6A and B). Conversely, the mean fluorescence intensity of GFAP in the SF/MXene + ES group was lower than that in the control (3.18), SF (2.88), and SF/MXene (1.79) groups.

Quantitative analysis showed that all the hydrogel groups favored axonal extension (Fig. 3B). The NSCs on SF/MXene hydrogel (76 ± 22.1 μm) extended much longer axons than those on SF hydrogel (26 ± 7.9 μm). The longest axons were observed in the SF/MXene hydrogel combined with the ES group (109 ± 10.6 μm), with a 320% improvement compared to the SF hydrogel. These results indicated that ES could further promote axonal extension and is beneficial to the construction of neuronal networks. In addition, we compared the axon density of each group, and the results showed the same trend as axon length (Fig. 3C). The direction of axon extension of neurons in each group was counted using rose wind diagrams. The results showed that the axon extension of neurons was undirected in the absence of an electric field. By comparison, the axons of neurons were able to extend in a certain direction under the guidance of the electric field, indicating that electric field stimulation has a certain directional effect on the extension of neuronal axons (Fig. 3D). The mRNA expression results of differentiation-related genes in each group generally aligned with the trend observed in immunofluorescence staining results (Fig. 3E and F). Specifically, the combination of ES and SF/MXene conductive hydrogel demonstrated a promotion in the differentiation of NSCs into neurons while reducing their differentiation into astrocytes. To further investigate the molecular mechanisms of SF/MXene hydrogel combined with ES that induces NSCs neural differentiation, we conducted an RNA sequencing analysis to identify relevant gene interactions. This analysis revealed 349 genes exhibiting differential expression, including 207 upregulated and 142 downregulated genes (Additional file 1: Fig. S7). These genes were further analyzed using Gene Ontology (GO) enrichment (Fig. 3G). For the Cellular Component (CC) category, the SF/MXene + ES group notably influenced various GO terms related to extracellular structures. This includes components of the extracellular region, membrane, and protein-containing complex. To elucidate the signal pathways influenced by the SF/MXene hydrogel combined with ES, we conducted an enrichment analysis using the Kyoto Encyclopedia of Genes and Genomes (KEGG) pathway database (Fig. 3H). The results revealed that pathways associated with neural differentiation, such as the Hippo signaling pathway, PI3K-Akt signaling pathway, and Wnt signaling pathway, were enriched. In summary, our observations suggested that the SF/MXene hydrogel combined with ES can induce NSCs to differentiate into neurons, forming neurites with certain directionality, through the regulation of specific signaling pathways.

ES can influence various physiological activities, including intracellular calcium channel expression, cell–matrix adhesion site arrangement, and growth factor receptor distribution, which regulate cellular physiological activities [45]. Previous studies have shown that simple conductive materials and ES can promote the differentiation of NSCs into neurons and inhibit astrocyte development in vitro [46]. The expression of voltage-gated calcium channels is upregulated after cells receive electrical signals and intracellular calcium levels are increased. The release of NO is catalyzed by nitric oxide synthase (NOS), which in turn activates cGMP and enhances the activity of growth factors, thus promoting cell proliferation. ES also promotes the differentiation of NSCs toward neurons by upregulating Ca_v_1 channel expression, which in turn enhances axon growth and extension [47, 48]. Our cell proliferation and differentiation results are consistent with the theory. These results indicate that the combined application of conductive hydrogel and ES can promote the differentiation of NSCs into neurons, and the presence of an electric field can also promote axon extension, both of which are beneficial for repair after traumatic brain injury.

Conventional theory suggests that the glial scar produced by activated astrocytes after TBI affects axonal regeneration and elongation [49]. Accordingly, we applied an electric field intensity of 300 mV cm^−1^ to SF/MXene hydrogel, the differentiation of NSCs to astrocytes was reduced, and the axons grew significantly after differentiation to neurons, with some directionality in the axon arrangement. Therefore, the combination of ES and conductive hydrogels for the regulation of NSCs differentiation is expected to achieve a superposition of dual advantages. Our results also revealed that SF/MXene conductive hydrogel promoted both NSCs differentiation into neurons and axon elongation, and this effect was further enhanced by the addition of ES.

### Validation of in vivo experiments for the TBI model

**Fig. 4:**
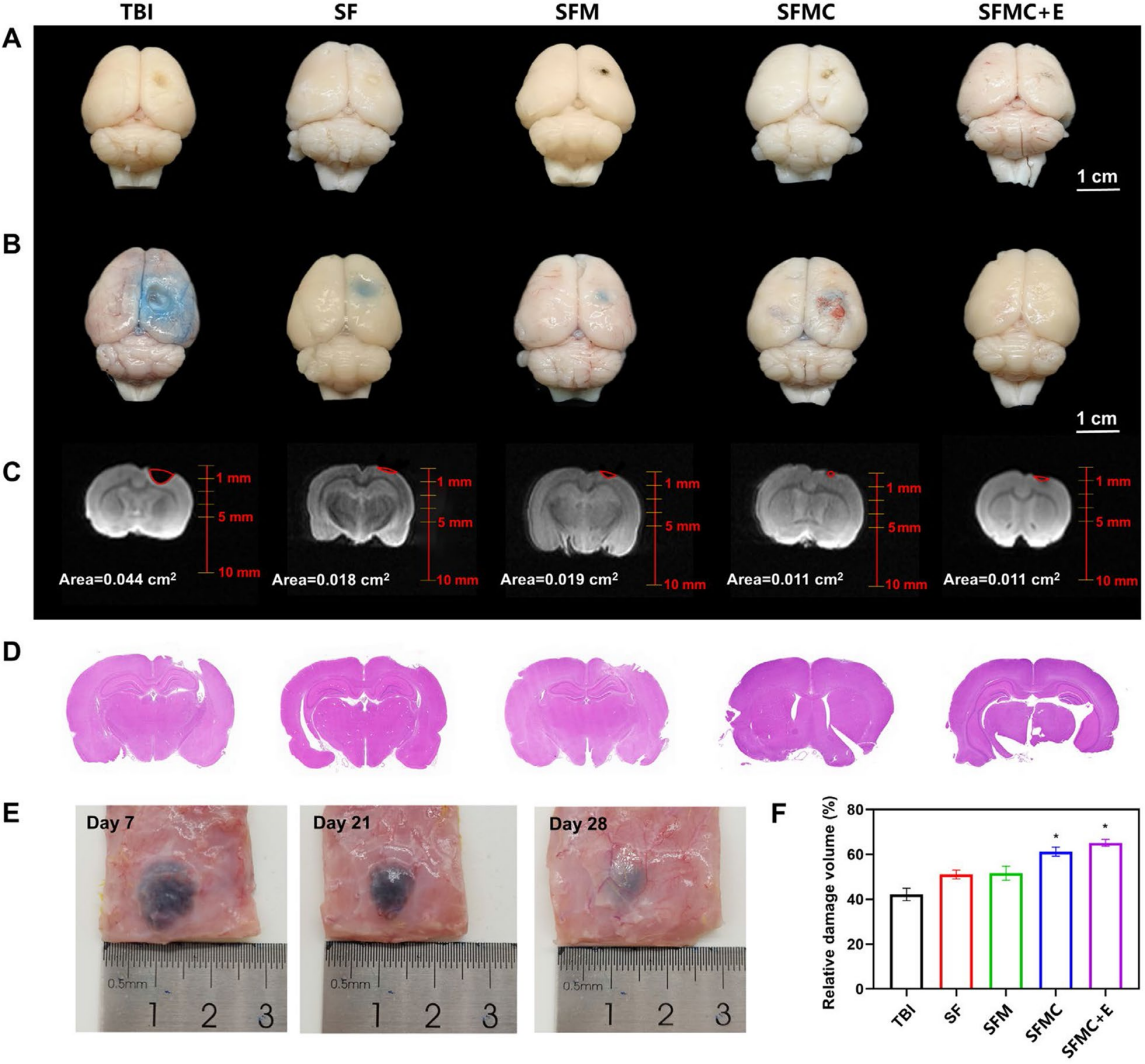
Evans blue staining, NMR imaging and H&E staining to evaluate lesion area after different treatment. **A** Digital images of various groups after TBI treatment. **B** Representative pictures of Evans blue extravasation in the right brains of various groups. **C** NMR imaging of TBI rat brain for analysis of lesion area. **D** H&E staining of lesion area. **E** Subcutaneously injected hydrogels on days 7, 21, and 28. **F** Quantitative analysis of the lesion volume. (± SD, n = 3; *P < 0.05)

Following 28 days of treatment, the TBI rats were euthanized, and their brains were extracted, sectioned, and subjected to specific staining and NMR analysis. Figure 4A presents the resulting brain images of various groups. We further investigate the effect of combined ES with SF/MXene hydrogel carrying NSCs in treating TBI. As shown in Fig. 4C, NMR imaging was used to examine the size of the cavity in the brain damage area of TBI rats. The results showed that after 28 d of treatment, the area of brain damage in all four groups of rats was significantly reduced. The addition of NSCs further reduced the cavity area, and the rats in the SFMC and SFMC + E groups showed better recovery than the rats in the hydrogel injection group alone. Quantifying the NMR results, Fig. 4F showed that the area of whole brain damage accounted for about 10% after TBI modeling. After 28 d of treatment, rats in the SFMC and SFMC + E groups recovered better and the cavity volume had significantly reduced.

H&E staining was used to evaluate the traumatic lesion cavity. As shown in Fig. 4D, the lesion area in the SFM hydrogel treatment group was reduced compared to the control group. SF/MXene hydrogel has a porous structure with a large pore size, which can promote the infiltration of nerve cells to a certain extent. Previous studies have shown that conductive hydrogels can activate endogenous NSCs neurogenesis in the lesion area, thus further promoting neural tissue regeneration in the damaged area [50]. We applied a similar method to validate the effectiveness. NMR results showed a reduced lesion area in the SFMC group. The SFMC + E group had the smallest lesion area, indicating that the combined therapy had the best therapeutic effect (Fig. 4F). After TBI, the integrity of the BBB is altered and subsequent brain edema causes secondary neurological damage. To determine whether the combination therapy could regulate the integrity and permeability of the BBB after TBI, we performed Evans blue staining. The results revealed that the SFMC + E group reduced the extent of BBB damage (Fig. 4B). As shown in Additional file 1: Fig. S8, the SFMC group, and SFMC + E group significantly reduced the extent of EB permeability and EB content in TBI rats compared with the control group. These results indicated that combination therapy could effectively maintain the integrity of the blood–brain barrier. Based on these results, we concluded that conductive hydrogel combined with NSCs and ES therapy could significantly improve the reconstruction of damaged brain tissues and have the best therapeutic effect on TBI. In order to meet the hydrogel is able to degrade at the right time period after implantation in the brain, we evaluated the degradation properties of the hydrogel in vivo (Fig. 4E). The SFM hydrogel was injected subcutaneously into the back of rats. Additional file 1: Fig. S9 showed that the implanted hydrogel gradually degraded and was able to stay in vivo for > 21 days. The degradation progress of the hydrogel matched the progress of TBI repair and was able to be replaced by newborn neural tissue at the appropriate time.

**Fig. 5:**
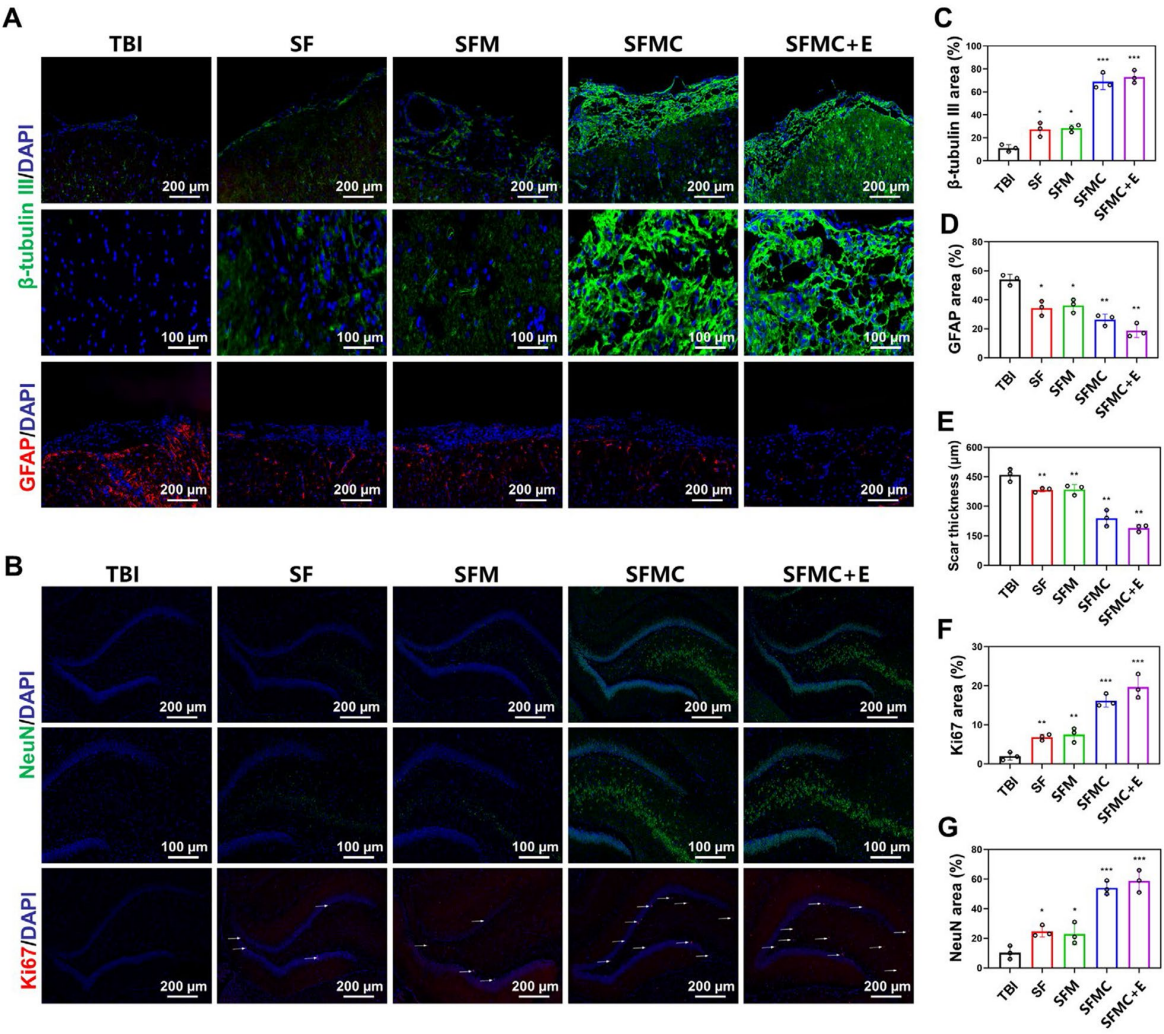
SF/MXene hydrogel combined ES can promote the differentiation of NSCs to neurons and can enhance hippocampal neurogenesis in TBI rats. **A** Investigation on NSCs differentiates into neurons and astrocytes. **B** Immunofluorescence to evaluate the neurogenesis in the DG zone. **C** Quantification of newborn neurons area (β-tubulin III), **D** astrocytes area (GFAP), and, **E** the scar thickness. **F** Quantification of mature neurons area (NeuN), **G** cell proliferation area (Ki67). (± SD, n = 5; *P < 0.05; **P < 0.01; ***P < 0.001)

To explore the differentiation of NSCs in the damaged region, we used immunofluorescence to assess the expression of β-tubulin III and GFAP. As shown in Fig. 5A, a small number of endogenous neuronal cells were visible in the TBI group, and the number of endogenous neuronal cells increased in the SF and SFM groups compared to the control group, but the difference was not significant. In the SFMC and SFMC + E groups, a large number of NSCs transplanted in the injury center differentiated into neurons and filled the injury area. The SFMC + E group had the highest percentage of positive neurons due to the presence of ES. Additionally, a large number of positive astrocytes were present in the periphery and center of the injury area in the TBI and SF groups. The number of astrocytes at the injury area was reduced in the SFM group after increasing the conductivity of the hydrogel and was mostly found around the injury. The SFMC group had a small number of astrocytes that might have been differentiated from NSCs, but the overall number of positive cells was still less than that of the SFM group. The SFMC + E group had the smallest percentage of positive GFAP area after adding ES to inhibit astrocyte differentiation. The statistical analysis of the positive area percentage of each group is shown in Fig. 5C and D. Glial scarring is an important factor that inhibits neuronal regeneration [51]. Therefore, overcoming the obstruction of glial scarring is important to promote neural regeneration and functional repair. As shown in Fig. 5E, the SFMC + E group had the smallest area of glial scar. To further assess the survival ability and proliferation of neurons in the DG region of TBI rats, we used immunofluorescence to assess neuronal nuclear antigen NeuN and proliferating cells (Ki67). The more NeuN-positive cells prove that there are more mature neurons in the DG area of rats. As illustrated in Fig. 5B, the positive expression of NeuN and Ki67 was significantly higher in the SFMC group and SFMC + E group compared to the TBI group, with even higher positive expression observed in the SFMC + E group. The quantitative analysis further confirmed the presence of more mature neurons and neoplastic cells in the rats treated with SFMC + E (Fig. 5F and G). Transplanted NSCs led to the increase of mature neurons in the DG area, and ES further increased the survival and proliferation of neurons.

### Behavioral recovery after TBI treatment

**Fig. 6:**
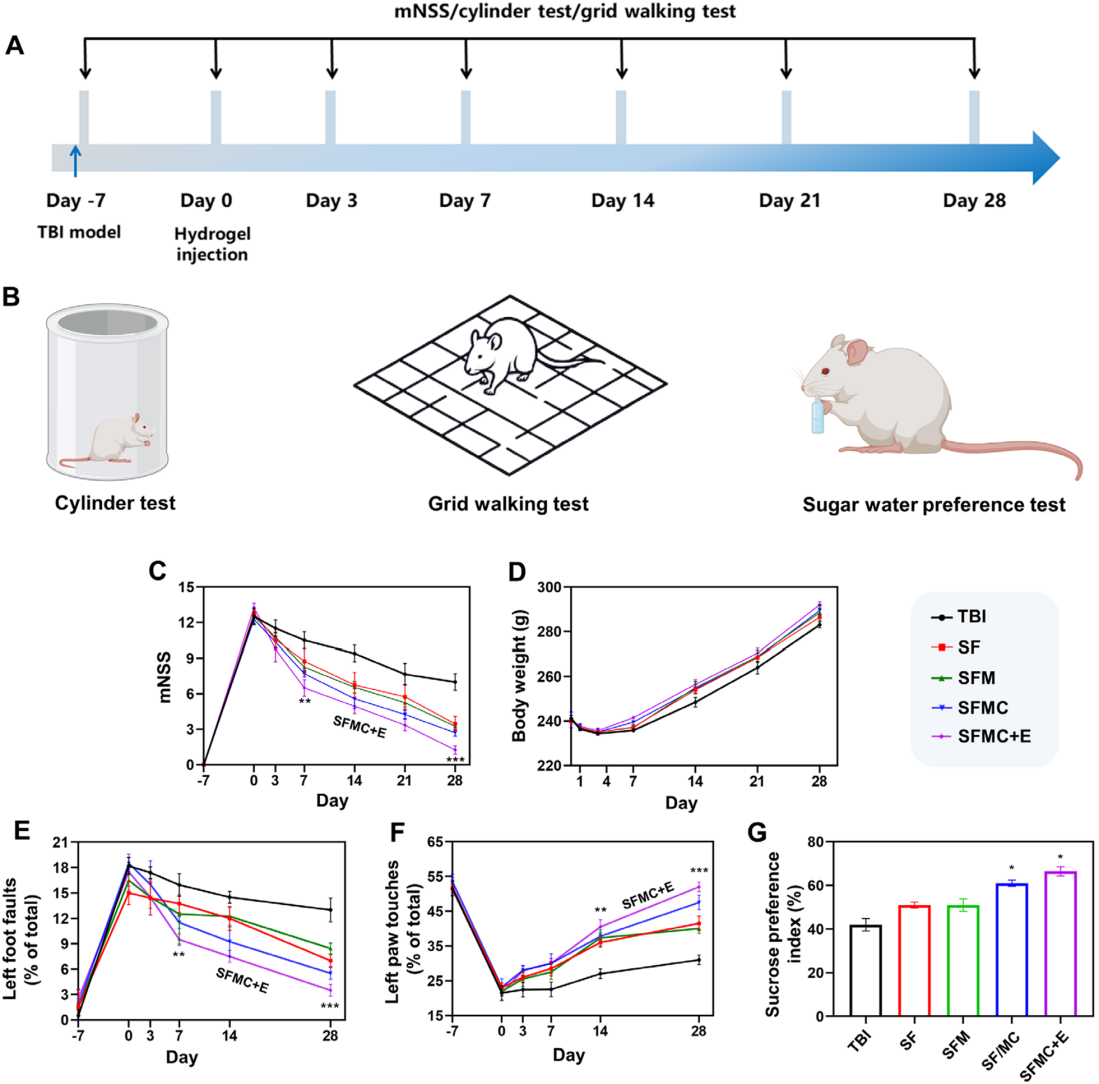
SF/MXene hydrogel combined ES can promote the behavioral recovery and emotional behaviors of TBI rats. **A** Experimental timeline of procedures and treatment administration in the TBI rats. **B** Schematic diagram of the cylinder test, grid walking test, and sugar water preference test. **C** mNSS scores for each group. **D** Weight change of each group. **E** Measurement of contralateral forelimb dexterity with the cylinder test. **F** Evaluation of the locomotor ability with the grid walking test. **G** Assessment of depression with the sucrose preference index. (± SD, n = 3; **P < 0.01; ***P < 0.001)

Figure 6A illustrates the experimental timeline for procedures, treatment administration, and behavioral testing. Motor coordination and mental status of the rats were assessed using the cylinder test, the grid walk test, and the sugar-water preference test (Fig. 6B). The general criterion for assessing behavioral recovery after TBI treatment is the use of scoring mechanisms. The mNSS test is a comprehensive assessment of the performance of sequelae symptoms in brain-injured animals, and we used this method [52]. As the time points after treatment were extended, the scores of all rat groups gradually decreased, indicating that neuromotor function was partially recovered in all groups. On days 7 and 14, the SFMC group and the SFMC + E group exhibited significantly lower mNSS scores compared with the TBI group (Fig. 6C). On days 21 and 28, there was a notable difference between the SFMC + E group and the TBI group. These findings indicate that the hydrogel combined with NSCs and ES therapy significantly promoted the recovery of neuromotor function in the early stage, with the combined treatment group demonstrating lower scores and better treatment effects in the later stage. The weight changes of the rats were also measured (Fig. 6D). Following TBI induction, the rats displayed symptoms of depression, significant weight loss, and reduced appetite and thirst. However, after 7 days of treatment, rats in all treatment groups ceased losing weight and gradually regained it. Furthermore, the mental status and activity of the rats in the combined treatment group were also better than those in the group treated with hydrogel alone throughout the treatment, which is consistent with the survival rate results. In the cylinder test, the percentage of wall touches by the affected forelimbs of rats in the TBI group was significantly reduced (Fig. 6E). This behavioral asymmetry was reversed when ES treatment was combined with the injection of conductive hydrogels carrying NSCs. During the grid walking test, the number of error steps was significantly increased in the affected hind limbs of TBI rats that fell under the grid (Fig. 6F). Notably, the number of forelimb touches and the number of foot errors exhibited recovery in the SFMC + E group compared to the TBI group. The video showed that the SFMC + E group had a better balance compared to the TBI group (Additional file 1: Videos S1 and S2). These results showed that the combination of ES with conductive hydrogel and NSCs had the best effect in promoting motor function recovery.

## Conclusion

In summary, we have successfully developed a novel ES therapy hydrogel system for TBI, the treatment showed high efficiency in *in-vitro* experiments, and the *in-vivo* efficiency is proved by the behavioral recovery of TBI rats. We conclude that MXene nanosheets incorporated in SF hydrogel combine the advantage of injectability, ECM-like mechanical properties, good biocompatibility, and high electrical conductivity, and are excellent carriers for NSCs transplantation to treat brain injury. This system overcomes the difficulty in the promotion of neuron differentiation and inhibition of astrocyte differentiation. It further produced longer, denser axons. Such practice showed great promise in animal experiments and provided guidance for future neural tissue engineering and pioneering stem cell therapy against TBI.

### Supplementary Information

**Additional file 1: Table S1.** The content of titanium (Ti) in each group of SF/MXene hydrogels. **Figure S1.** Young’s modulus of SF-based hydrogels with varying MXene content after injection with a 26-gauge needle. **Figure S2.** Loss modulus G’’ of SF-based hydrogels with varying MXene content. **Figure S3.** The swelling of SF-based hydrogels with varying MXene content. **Figure S4.** Percentage of viable cells in each group. Figure S5. Proportions of neurons (**A**) and glial cells (**B**) in each group on days 3 and 10. **Figure S6.** Quantitative analysis of fluorescence intensity of β-tubulin III-positive cells (**A**) and GFAP-positive cells (**B**). **Figure S7.** Volcano plot depicting expression level variations between the SF/MXene + ES and SF groups. Red dots illustrate significantly upregulated genes, green dots indicate notably downregulated genes and gray dots signify genes with no significant differential expression. **Figure S8.** Quantification of Evans blue was tested by the spectrophotometry after the brain tissues were lysed. **Figure S9.** In vivo degradation curve of the implanted SF/MXene hydrogel. **Table S2.** The sequences of primers used for the qPCR analysis. Video S1. Performance of rats in the TBI group on the balance beam. Video S2. Performance of rats in the SFMC + E group on the balance beam.

## Data Availability

No datasets were generated or analysed during the current study.
