# Supplementary material for: Injectable Bombyx mori (B. mori) silk fibroin/MXene conductive hydrogel for electrically stimulating neural stem cells into neurons for treating brain damage
Source: J Nanobiotechnology. 2024 Mar 14;22:111. doi: 10.1186/s12951-024-02359-x (PMC10941401; doi:10.1186/s12951-024-02359-x)
Supplement: Supplementary file 1 — Additional file 1: Table S1. The content of titanium (Ti) in each group of SF/MXene hydrogels. Figure S1. Young’s modulus of SF-based hydrogels with varying MXene content after injection with a 26-gauge needle. Figure S2. Loss modulus G’’ of SF-based hydrogels with varying MXene content. Figure S3. The swelling of SF-based hydrogels with varying MXene content. Figure S4. Percentage of viable cells in each group. Figure S5. Proportions of neurons (A) and glial cells (B) in each group on days 3 and 10. Figure S6. Quantitative analysis of fluorescence intensity of β-tubulin III-positive cells (A) and GFAP-positive cells (B). Figure S7. Volcano plot depicting expression level variations between the SF/MXene + ES and SF groups. Red dots illustrate significantly upregulated genes, green dots indicate notably downregulated genes and gray dots signify genes with no significant differential expression. Figure S8. Quantification of Evans blue was tested by the spectrophotometry after the brain tissues were lysed. Figure S9. In vivo degradation curve of the implanted SF/MXene hydrogel. Table S2. The sequences of primers used for the qPCR analysis. Video S1. Performance of rats in the TBI group on the balance beam. Video S2. Performance of rats in the SFMC + E group on the balance beam. [file 12951_2024_2359_MOESM1_ESM.docx]

**Additional file**

**Injectable *Bombyx mori* (*B.mori*) silk fibroin/MXene conductive hydrogel for electrically stimulating neural stem cells into neurons for treating brain damage**

Zhangze Yang^†^, Yuxin You^†^, Xiangyu Liu, Quan Wan, Zongpu Xu, Yajun Shuai, Jie Wang, Tingbiao Guo, Jiaqi Hu, Junhui Lv, Meng Zhang, Mingying Yang^*^, Chuanbin Mao^*^, Shuxu Yang^*^

^†Zhangze Yang and Yuxin You contributed equally to this work^

^*Corresponding author: Mingying Yang Email: yangm@zju.edu.cn^

^*Corresponding author: Chuanbin Mao Email: maophage@gmail.com^

^*Corresponding author: Shuxu Yang Email: 3195019@zju.edu.cn^

**Authors’ affiliations**

1. Institute of Applied Bioresource Research, College of Animal Science, Key Laboratory of Silkworm and Bee Resource Utilization and Innovation of Zhejiang Province, Hangzhou, Zhejiang, 310058, China.

Zhangze Yang, Quan Wan, Zongpu Xu, Yajun Shuai, Jie Wang, Jiaqi Hu, Meng Zhang, Mingying Yang

1. Department of Neurosurgery, Sir Run Run Shaw Hospital, School of Medicine, Zhejiang University, Hangzhou, 310016, China.

Junhui Lv, Shuxu Yang

1. Institute of Biotechnology, College of Agriculture and Biotechnology, Zhejiang University, Hangzhou, Zhejiang 310058, China.

Yuxin You

1. School of Materials Science and Engineering Zhejiang University, Hangzhou, Zhejiang 310027, China.

Xiangyu Liu

1. Centre for Optical and Electromagnetic Research National Engineering Research Center for Optical Instruments Zhejiang University Hangzhou 310058 China.

Tingbiao Guo

1. School of Materials Science and Engineering Zhejiang University, Department of Biomedical Engineering, The Chinese University of Hong Kong, Sha Tin, 999077, Hong Kong SAR P. R. China

Chuanbin Mao

**Experimental Section**

*Preparation of SF solution*

Our regenerated SF solution was extracted using the lithium bromide dissolution method. A total of 20 g of chopped silkworm cocoon shells were placed in a 0.02 mol·L^-1^ Na_2_CO_3_ boiling water solution and boiled for 30 minutes to remove the sericin on the surface of the cocoons. The degumming process was repeated twice, and the degummed SF fibers were cleaned with deionized water, air-dried naturally, and set aside. A 100 ml solution of 9.3 mol·L^-1^ LiBr was prepared, and 10 g of the degummed SF fibers were dissolved in the LiBr solution. Subsequently, the solution was transferred to a dialysis bag (molecular weight cut-off 8000-14000 Da) and dialyzed in deionized water for 3 d to remove the salt ions from the solution. The water was changed every 2-3 h during dialysis to ensure complete dialysis. After dialysis, the SF solution was centrifuged to remove impurities under the conditions of 3000 rpm for 10 minutes. Finally, the supernatant was collected, and the concentration of silk fibroin protein was measured by the gravimetric method and stored at 4°C for use in the preparation of hydrogels.

*Fourier Transform Infrared Analysis*

The structural characteristics of SF/MXene hydrogels and SF hydrogel were analyzed using Fourier transform infrared (FTIR) spectroscopy. The hydrogel samples were scanned using an FTIR spectrometer (FTIR-8400s, SHIMADZU) in the range of 4000-400 cm^−1^, with a resolution of 4 cm^-1^ and 40 scans.

*Morphological Observation*

The morphology of SF/MXene hydrogels and SF hydrogel was observed by using a scanning electron microscope instrument (SEM, TM-1000, Hitachi). Briefly, the hydrogels were frozen in liquid nitrogen and placed at -80℃ for 2 h and lyophilized using a freeze dryer for 48 h. The freeze-dried samples were then cut to obtain cross-sectional views of the hydrogels. Before testing, the samples were coated with a layer of gold.

*Swelling Behavior*

The swelling ratio of SF/MXene hydrogels and SF hydrogels was calculated using the following equation: Swelling (%) = (M_S_-M_L_) / M_L_×100%. Briefly, the lyophilized hydrogels were weighed (M_L_) and placed in PBS (pH=7.2~7.4). At specific time points, the samples were removed from the PBS solution, and the water on the surface of the hydrogels was quickly dried with filter paper. The swollen hydrogels were then weighed (M_S_).

*Rheological Test*

The rheological characterization of SF/MXene hydrogels and SF hydrogel was performed using a rotational rheometer (MCR302, Antompa Corporation, USA) at 25 °C. The rheometer was equipped with a parallel diameter of 15 mm. Typically, the hydrogel with 15 mm diameter and 1 mm height was measured in a constant strain mode of 1% with a frequency range from 0.1~100 Hz. To assess the injectable behavior of the hydrogels, angular frequency sweep measurements (0.1~100 rad·s^-1^) were conducted at a fixed strain of 1%.

*Mechanical Property Test*

To evaluate the mechanical properties of SF/MXene hydrogels and SF hydrogels, we analyzed their compression capability of hydrogels using an electronic universal testing machine (AGS-J, SHIMADZU, USA). For the compression test, the cylindrical hydrogels with a diameter of 10 mm and a height of 5 mm were prepared and measured by a 5 N sensor. The hydrogels’ elastic modulus, maximum stress, and maximum strain were measured. Each sample was tested in triplicate.

*Electrical Performance Test*

The conductivity of the SF/MXene hydrogels was calculated by multimeter according to the following equations:

R=ρ×L/S (1) σ=1/ρ (2)

Where R is the resistance, L is the length of the hydrogel, S is the cross-sectional area of the hydrogel, ρ is the resistivity and σ is the conductivity. Equation (1) is the formula for resistance and resistivity, and Equation (2) shows that conductivity is the reciprocal of resistivity. The electrochemical impedance spectroscopy (EIS) measurements of SF/MXene hydrogels were performed using the CHI660C electrochemical workstation under open circuit conditions. The three-electrode system consisted of a pure platinum sheet as the counter electrode, an Ag/AgCl electrode as the reference electrode, and 0.067 M PBS solution as the electrolyte solution. The frequency range is 10^-2^ Hz to 10^5^ Hz, and the compensation amplitude is 5 mV. The Alternating Current (AC) impedance of the hydrogels was measured, and the corresponding AC impedance curve was obtained.

Supplementary Information

Table S1. The content of titanium (Ti) in each group of SF/MXene hydrogels.


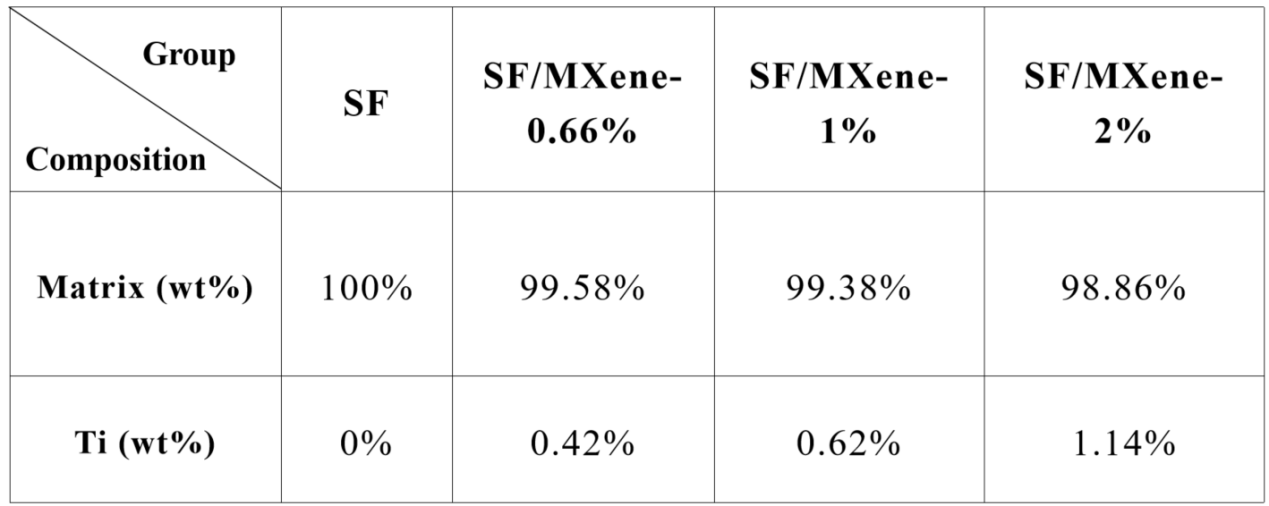


**
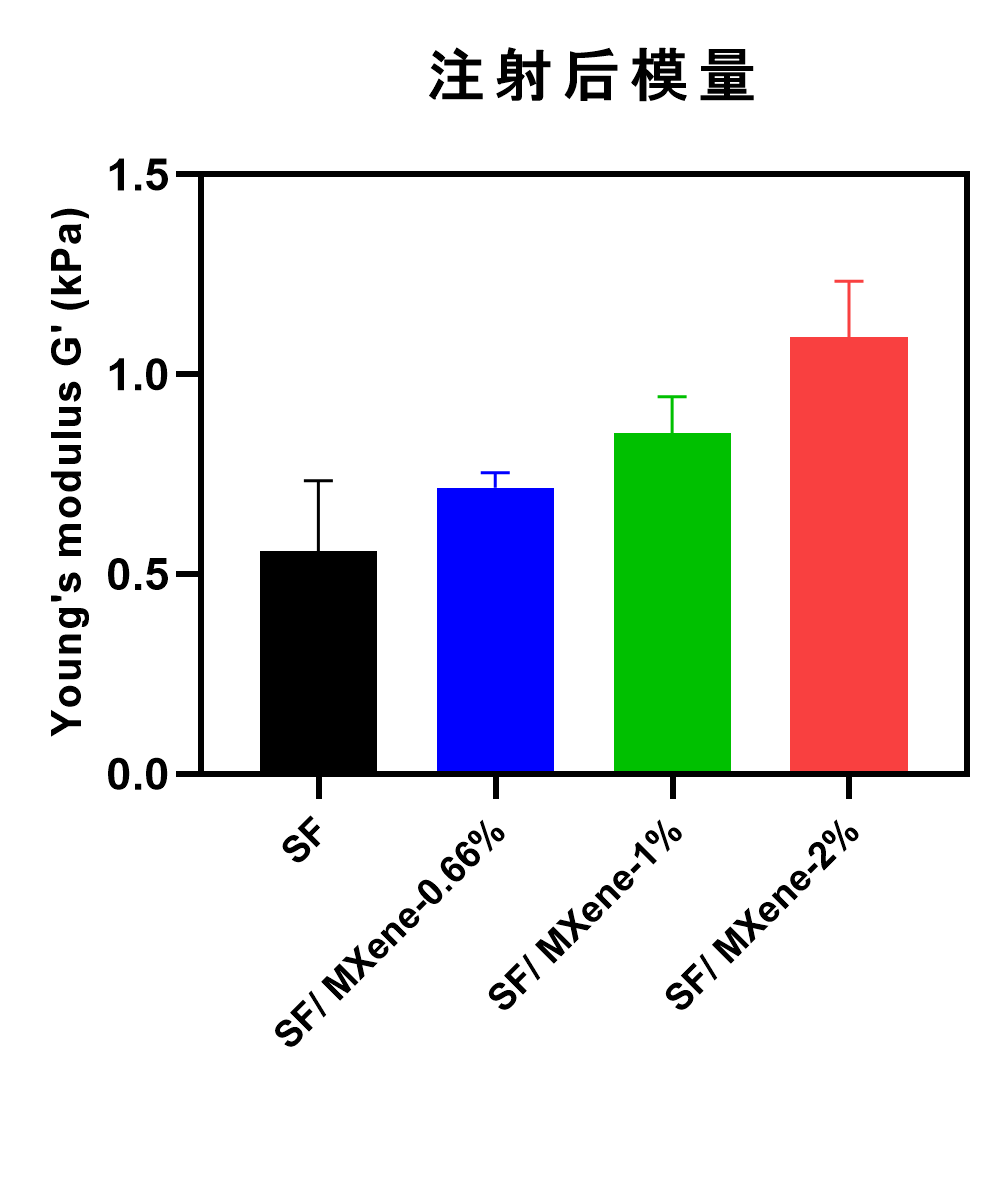
**

**Figure S1.** Young’s modulus of SF-based hydrogels with varying MXene content after injection with a 24G needle.


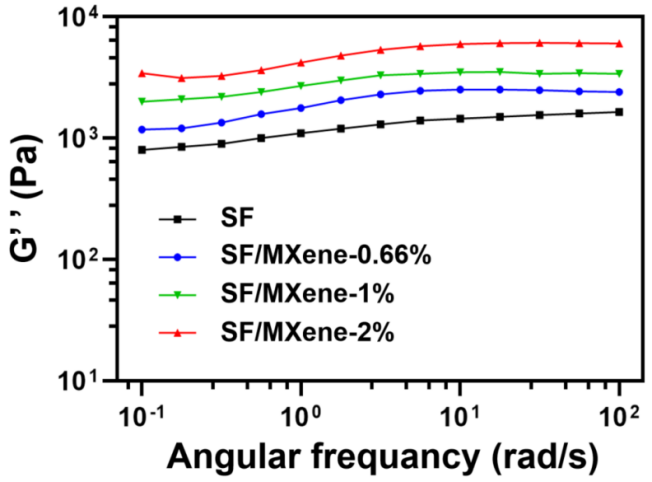


**Figure S2.** Loss modulus G’’ of SF-based hydrogels with varying MXene content.


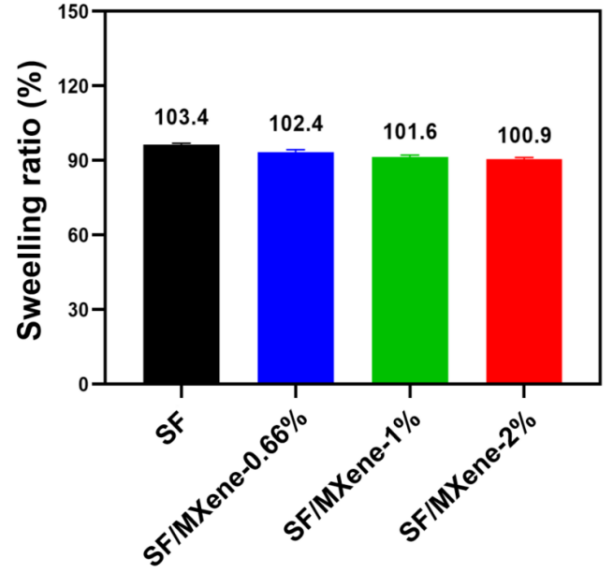


**Figure S3.** The swelling of SF-based hydrogels with varying MXene content.


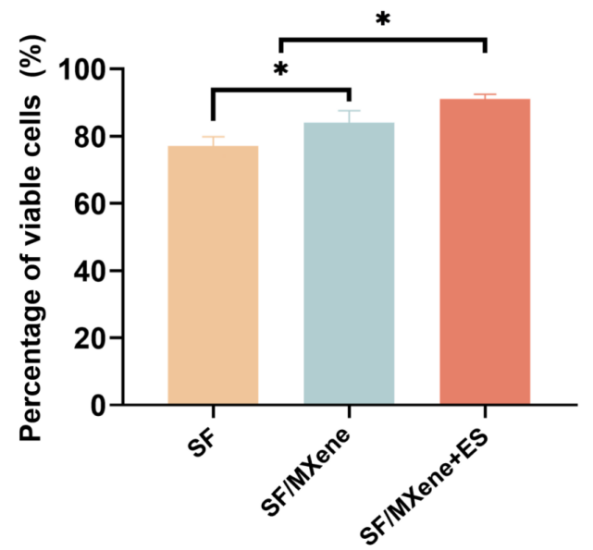


**Figure S4.** Percentage of viable cells in each group.

**
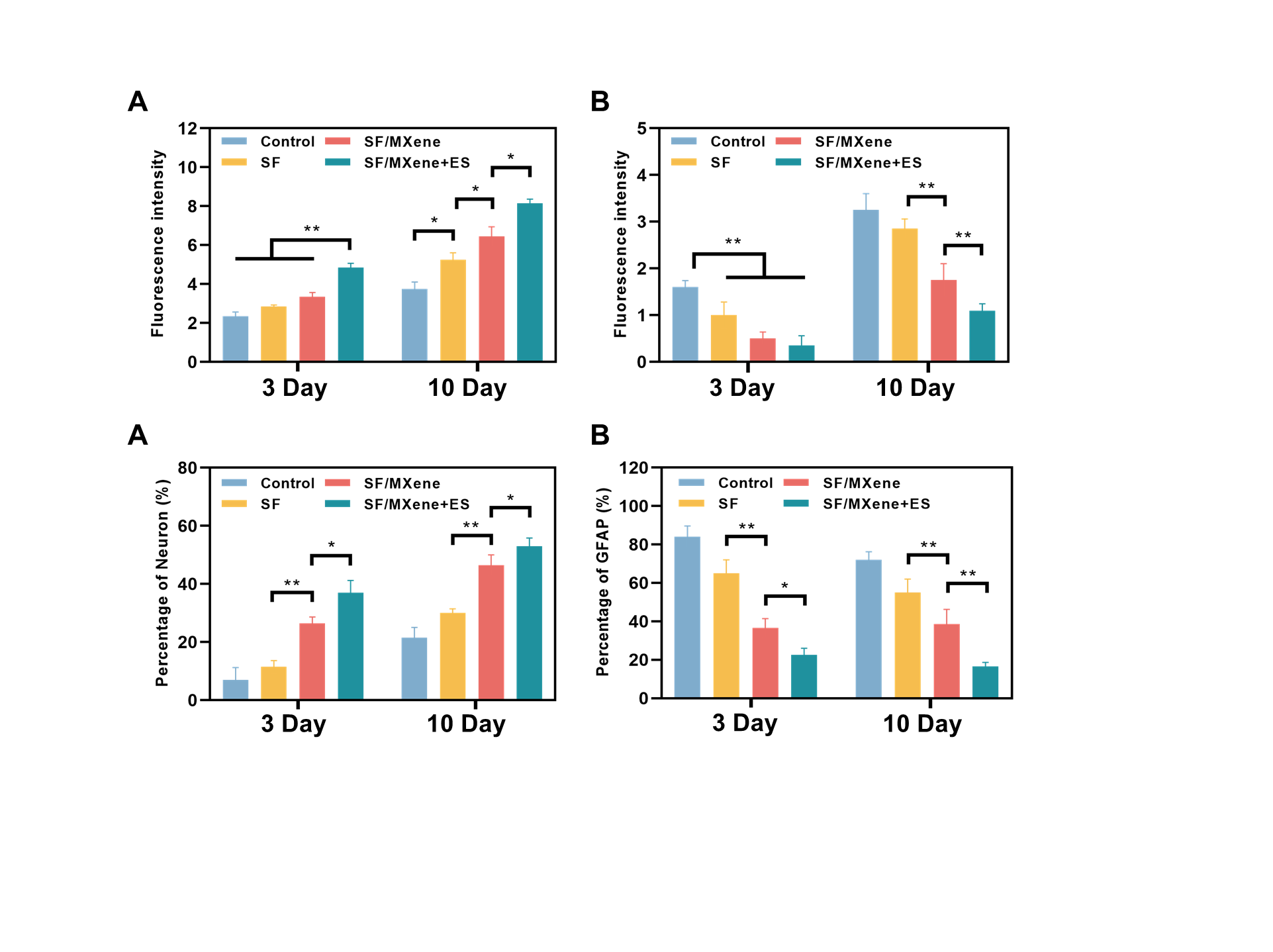
**

**Figure S5.** Proportions of neurons (A) and glial cells (B) in each group on days 3 and 10.

**
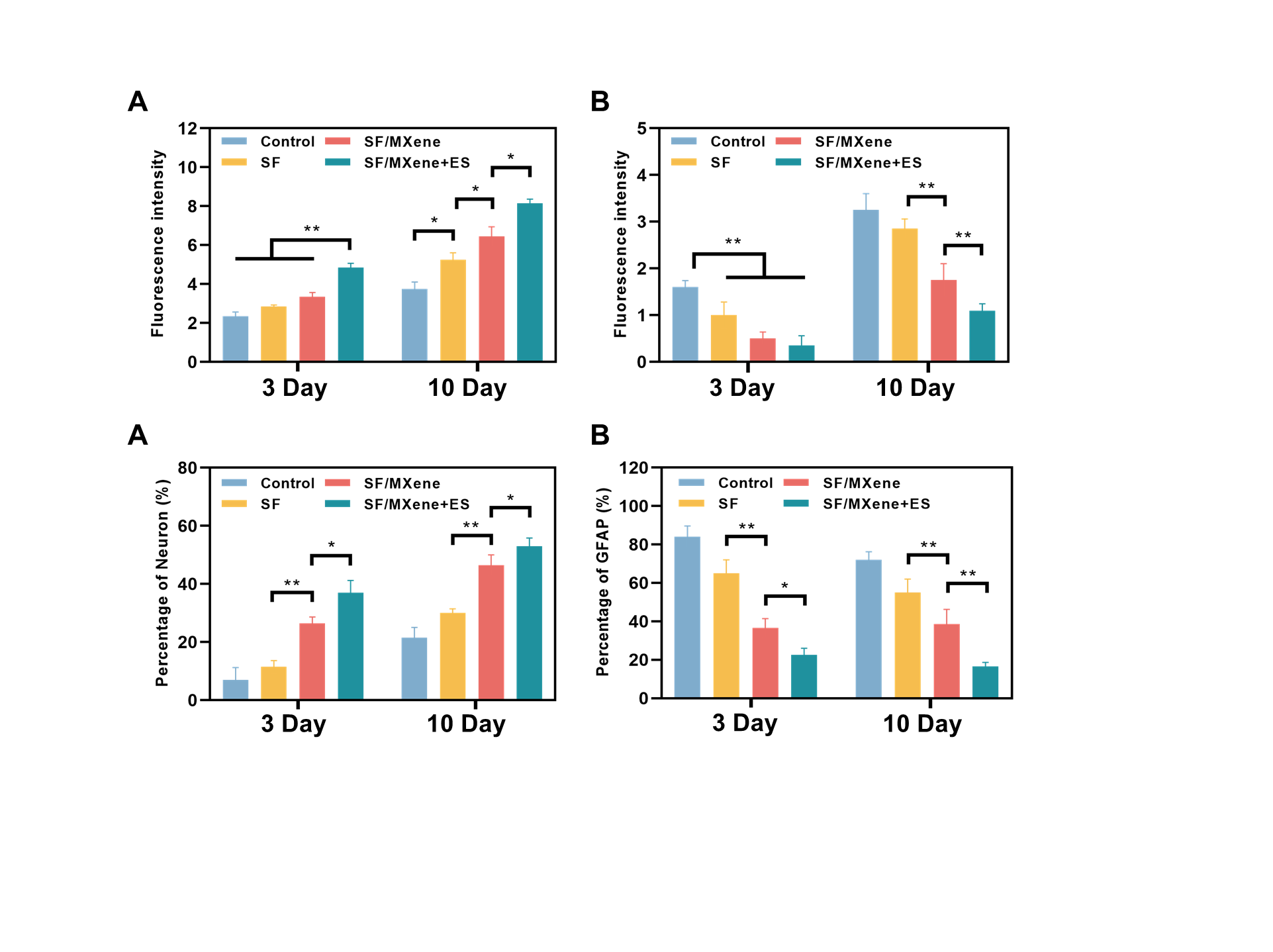
**

**Figure S6.** Quantitative analysis of fuorescence intensity of β-tubulin III-positive cells (A) and GFAP-positive cells (B).


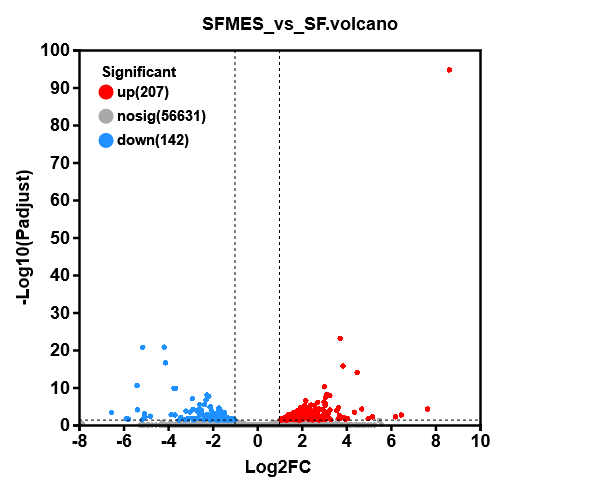


**Figure S7.** Volcano plot depicting expression level variations between the SF/MXene + ES and SF groups. Red dots illustrate significantly upregulated genes, green dots indicate notably downregulated genes, and gray dots signify genes with no significant differential expression.

**
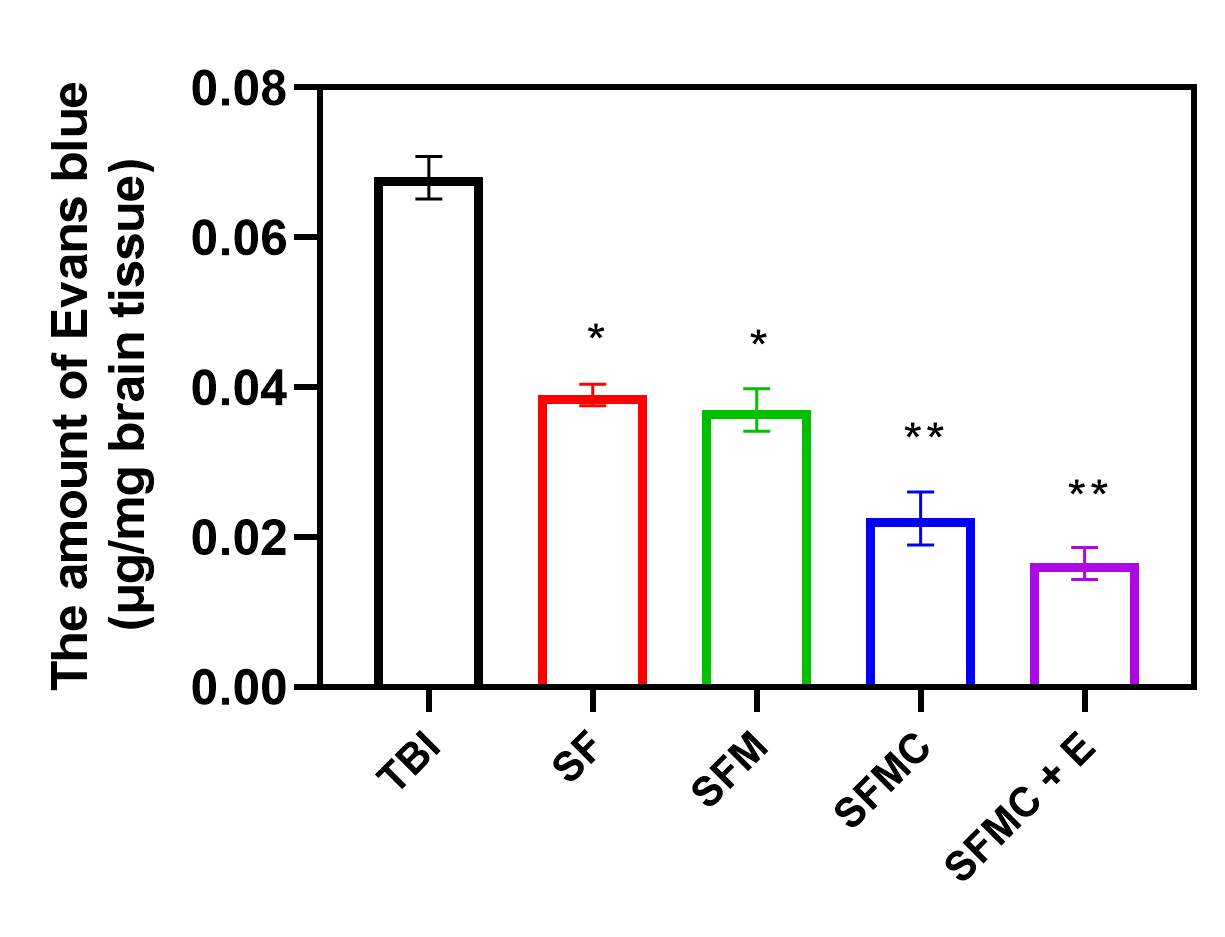
**

**Figure S8.** Quantification of Evans blue was tested by the spectrophotometry after the brain tissues were lysed.


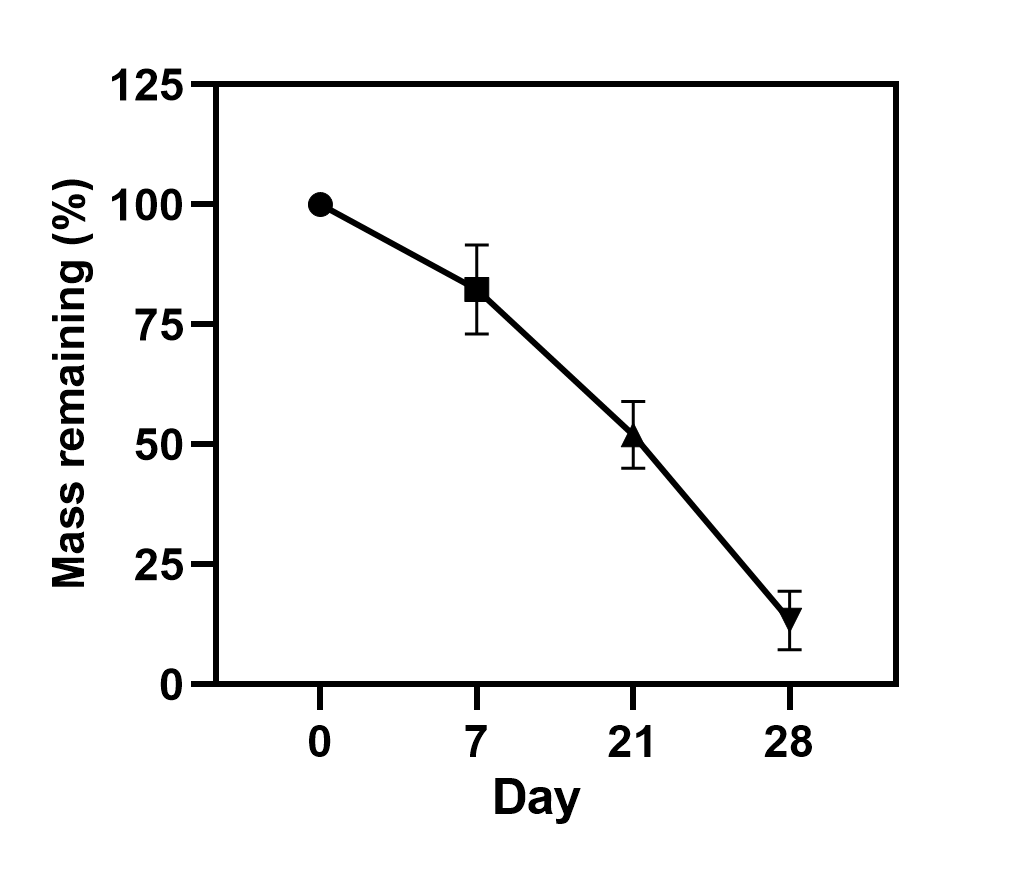


**Figure S9.** *In Vivo* degradation curve of the implanted SF/MXene hydrogel.

**Table S2.** The sequences of primers used for the qPCR analysis.

| Gene | Forward prime | Reverse primer | Length (bp) |
| --- | --- | --- | --- |
| β tubulin-III | CCAGTGCGGCAACCAGATAG | GGCTCTGGGCACATACTTGTGA | 160 |
| GFAP | AGTGGCCACCAGTAACATGCAA | GGACTCAAGGTCGCAGGTCAA | 163 |

**Video S1.** Performance of rats in the TBI group on the balance beam.

**Video S2.** Performance of rats in the SFMC + E group on the balance beam.
